# Genetic Control of Vulval Development in *Caenorhabditis briggsae*

**DOI:** 10.1534/g3.112.004598

**Published:** 2012-12-01

**Authors:** Devika Sharanya, Bavithra Thillainathan, Sujatha Marri, Nagagireesh Bojanala, Jon Taylor, Stephane Flibotte, Donald G. Moerman, Robert H. Waterston, Bhagwati P. Gupta

**Affiliations:** * Department of Biology, McMaster University, Hamilton, Ontario L8S 4K1, Canada; † Michael Smith Laboratories, University of British Columbia, Vancouver, British Columbia V6T 1Z4, Canada; ‡ Department of Zoology, University of British Columbia, Vancouver, British Columbia V6T 1Z4, Canada; § Department of Genome Sciences, University of Washington, Seattle, Washington 98195-5065

**Keywords:** *C. briggsae*, *C. elegans*, vulva, development, cell proliferation, differentiation, morphogenesis, egg-laying defective

## Abstract

The nematode *Caenorhabditis briggsae* is an excellent model organism for the comparative analysis of gene function and developmental mechanisms. To study the evolutionary conservation and divergence of genetic pathways mediating vulva formation, we screened for mutations in *C. briggsae* that cause the egg-laying defective (Egl) phenotype. Here, we report the characterization of 13 genes, including three that are orthologs of *Caenorhabditis elegans unc-84* (SUN domain), *lin-39* (*Dfd/Scr*-related homeobox), and *lin-11* (LIM homeobox). Based on the morphology and cell fate changes, the mutants were placed into four different categories. Class 1 animals have normal-looking vulva and vulva-uterine connections, indicating defects in other components of the egg-laying system. Class 2 animals frequently lack some or all of the vulval precursor cells (VPCs) due to defects in the migration of P-cell nuclei into the ventral hypodermal region. Class 3 animals show inappropriate fusion of VPCs to the hypodermal syncytium, leading to a reduced number of vulval progeny. Finally, class 4 animals exhibit abnormal vulval invagination and morphology. Interestingly, we did not find mutations that affect VPC induction and fates. Our work is the first study involving the characterization of genes in *C. briggsae* vulva formation, and it offers a basis for future investigations of these genes in *C. elegans*.

Invertebrate model organisms such as the nematode *Caenorhabditis elegans* are excellent model organisms for investigating the genetic basis of development. Studies in *C. elegans* have provided insights into the cellular and molecular basis of organ formation and have revealed similarities and differences in the formation of homologous structures in metazoans.

Nematodes are an attractive system for studying the evolution of developmental mechanisms because they offer many useful features, including rapid development, transparency, and large brood size. Comparative studies in nematodes have revealed similarities and differences in the vulva, the egg-laying organ. For example, the vulval precursor cell (VPC) equivalence group in *Oschieus tipulae* and *Pristionchus pacificus* is smaller than that of *C. elegans* (Sommer 2005; Sternberg 2005). Furthermore, in *Pristionchus*, the mechanism of restricting vulval precursor competence is different. Although cell fusion limits precursor competence in *C. elegans*, programmed cell death controls this process in *P. pacificus* (Sommer 2005). In addition to these two species, vulval morphology has been examined in a large number of other nematodes, and differences have been found in the number of vulval progeny and the placement of the vulva (Felix *et al.* 2000; Felix and Sternberg 1997; Sommer *et al.* 1994; Sommer and Sternberg 1996). More recently, Kiontke *et al.* (Kiontke *et al.* 2007) examined 51 rhabditid species and identified variations in different steps of vulva development. In the *Caenorhabditis* genus, *Caenorhabditis briggsae* is an excellent model for comparative and evolutionary studies (Gupta *et al.* 2007). Sequence analyses of *C. elegans* and *C. briggsae* have suggested a divergence of approximately 30 million years (Cutter 2008). Morphologically, *C. briggsae* is almost identical to *C. elegans*; however, sequence comparison has revealed that almost one-third of all predicted genes in its genome are highly divergent (Gupta and Sternberg 2003; Stein *et al.* 2003). Both organisms offer powerful tools for dissecting gene function, including rapid development, invariant cell lineages, fully sequenced genomes, and amenability to both genetic and molecular manipulation (Antoshechkin and Sternberg 2007; Gupta *et al.* 2007; Hillier *et al.* 2007; Stein *et al.* 2003; Zhao *et al.* 2010). The hermaphroditic mode of reproduction of these species is another advantage because it allows for the maintenance of mutations that affect mating and egg laying. Organisms with divergent genomes but overall morphological similarity may offer intriguing examples of how networks of genes can be regulated differently while yielding the same ultimate structure.

Comparative studies of *C. elegans* and *C. briggsae* have revealed that alterations in developmental mechanisms do not always affect morphology. For example, the expression pattern of *lin-39*, an important Hox family member (*Dfd/Scr*-related) that regulates VPC competence, differs between the two species, yet VPC induction and cell fates are conserved (Penigault and Felix 2011a). The role of the Wnt pathway effector *pop-1* (TCF/LEF family) in *C. briggsae* endomesoderm specification represents yet another case of altered gene function with no obvious change in embryonic cell divisions or tissue morphology (Lin *et al.* 2009). In another case, knockdown of the *lin-12/Notch* receptor family member *glp-1* causes a multivulva (Muv) phenotype in *C. briggsae* but not in *C. elegans* (Rudel and Kimble 2001). Thus, *glp-1* appears to have acquired a new function in negative regulation of VPC fate specification in *C. briggsae*. Such alterations in gene function without apparent changes in homologous characters were described originally as developmental system drift (DSD) (True and Haag 2001).

The egg-laying system of *C. briggsae* is well suited for comparative analysis of gene function in organ formation, and it is helpful in elucidating DSD. Morphologically, the system is identical to *C. elegans* and follows a similar sequence of developmental events. Several of the vulval characters, such as cell number, position in the midbody region, and cell fusions are shared between these two species. However, some differences also have been noted. For example, the division frequency of the P3.p vulval precursor is higher in *C. elegans* than in *C. briggsae* (Delattre and Felix 2001). Other differences that have been found include the role of anchor cell (AC) in the vulval induction process, uterine seam (utse) cell morphology, brood size, sheath-contraction rate, and reproductive efficiency (Delattre and Felix 2001; Felix 2007; Gupta and Sternberg 2003; Miller *et al.* 2004). Subtle variations in VPC responses to inductive and lateral signaling cascades also have been reported (Felix 2007; Hoyos *et al.* 2011). Thus, there are some distinct differences in the mechanisms of vulva formation and egg-laying between *C. elegans* and *C. briggsae*.

In *C. elegans*, the egg-laying system is composed of five different cell types, namely, the vulva, somatic gonad (uterus), vulva and uterine muscles, and neurons (Li and Chalfie 1990). The vulva is connected to the uterus via a multinucleated utse cell (Newman and Sternberg 1996) and serves as a passageway for egg laying. Defects in any of the egg-laying components can cause eggs to accumulate in the uterus, resulting in an Egg-laying defective (Egl) phenotype. The *C. elegans* vulva is formed by the descendants of three of six equipotent VPCs. The VPCs are the posterior daughters of P cells. At hatching, the L1 larva contains six bilaterally symmetrical pairs of P cells in the ventrolateral region. By the mid-L1 stage, P cells migrate into the ventral cord region and become arranged in a single row (numbered P1 to P12) (Sulston 1976 and Sulston and White 1980). This process involves an orchestrated series of events initiated by the directed migration of P-cell nuclei. As a nucleus migrates, it drags the rest of the cell body along with it. Several genes have been identified that affect P-cell nuclear migration, including UNC-83 (KASH domain) and UNC-84 [SUN domain (Starr 2011)]. These two proteins are localized to the outer and inner nuclear membranes, respectively (McGee *et al.* 2006), and they bridge the nuclear envelope and facilitate nuclear migration by transferring forces from the cytoskeleton to the nuclear lamina.

Soon after arriving at the ventral cord region, all 12 P cells divide once along the anteroposterior axis. Of the posterior daughters, five (P1.p, P2.p, P(9-11).p) fuse with the hyp7 syncytium during the L1 stage. P12.p produces two daughters: P12.pp, which undergoes programmed cell death, and P12.pa, which adopts a unique epidermal fate, hyp10. The remaining 6 Pn.p cells (n = 3 to 8, VPCs) remain unfused in L1 due to the action of the Hox gene *lin-39*. These VPCs respond to later developmental cues. P3.p loses competence in the L2 stage in roughly half of animals and fuses with hyp7 (termed ‘F’ fate).

Although all six VPCs are equally capable of giving rise to vulval tissue, only P5.p, P6.p, and P7.p do so in wild-type animals. This is due to the action of three evolutionarily conserved signal transduction pathways mediated by LET-60/Ras-MPK-1/MAPK (inductive signaling), LIN-12/Notch (lateral signaling), and Wnt-BAR-1/β-catenin (Eisenmann 2005; Greenwald 2005; Sternberg 2005). In the L3 stage, the gonadal AC secretes the ligand LIN-3/EGF that binds to LET-23/EGFR on VPCs, leading to the activation of LET-60/Ras signaling in P6.p and to a lesser extent in P5.p and P7.p. Induced P6.p serves as a source of lateral signal that activates the LIN-12/Notch receptor in P5.p and P7.p. The inductive and lateral signaling together specify 1° (P6.p) and 2° (P5.p and P7.p) cell fates. In addition, Wnt signaling also participates in this process (Gleason *et al.* 2006; Seetharaman *et al.* 2010). The remaining uninduced VPCs fuse with hyp7 after one cell division (termed 3° fate).

Forward genetics is an elegant method by which to study vulva formation in *C. briggsae* and to compare its developmental mechanisms with *C. elegans*. We have isolated mutations in *C. briggsae* AF16 by using the Egl phenotype as an assay. In this study, we describe 19 mutants, 17 of which fall into four phenotypic categories and represent 13 different genes. Class 1 mutants exhibit the Egl phenotype with normal vulval cells and morphology. Class 2 mutants lack some or all of the Pn.p cells in the ventral hypodermal region, suggesting that these genes play important roles in maintaining the correct number of P cells in the ventral hypodermal region. Class 3 mutants have a normal number of VPCs, but some precursor cells fail to be induced. Class 4 mutants affect the differentiation of vulval progeny and lead to abnormal vulval morphology in L4 larvae and a protruding vulva (Pvl) phenotype in adults. We also provide evidence that three of the genes recovered in our screen, *Cbr-lin(sy5506)* (class 2), *Cbr-lin(bh20)* (class 3), and *Cbr-lin(sy5336)* (class 4), are orthologs of *C. elegans unc-84*, *lin-39*, and *lin-11*, respectively.

The mutants and phenotypic classes described here serve as the nucleus of our effort to investigate the genes involved in vulva formation in *C. briggsae*. In addition, they provide a tool for identifying interacting genes through enhancer and suppressor screens. These findings will facilitate the comparison of cellular and molecular processes between *C. briggsae* and *C. elegans* in studying conservation and divergence in developmental mechanisms.

## Materials and Methods

### Strains and culture conditions

Wild-type *C. briggsae* AF16 was used as a reference strain in all experiments. Strains were maintained at 20° using culture methods described for *C. elegans* (Brenner 1974; Wood 1988). To obtain synchronized animals, gravid hermaphrodites were bleached. The bleach solution was prepared using sodium hypochlorite (commercial bleach) and 4 N sodium hydroxide (NaOH) at a ratio of 3:2. For 2 volumes of worms washed with M9 buffer, 1 volume of the bleach solution was added. The solution was vortexed and left to stand for 3 min at room temperature. After three consecutive washes with M9 solution, a pellet with 1 mL of remaining M9 buffer was transferred to an Eppendorf tube and placed in a shaker. Twenty-four hours later, the F1 worms were plated onto a new NG plate.

The strains used in this study are listed below (linkage groups of mapping markers are also mentioned; see www.briggsae.org for details). The *egl(bh6)* strain [allelic to *egl(bh2)*] was lost during the course of this study. The ‘Cbr’ prefix denotes the *C. briggae* orthologs of known *C. elegans* genes.

Mapping mutants: *dpy(sy5148) II*, *dpy(sy5022) III*, *sma(sy5330) I*, *unc(s1270) IV*, *unc(sa997) V*, *unc(sy5077) X*.
Egl and Vul mutants: *egl(bh2)*, *egl(bh6)*, *egl(bh21)*, *egl(sy5395)*, *lin(bh7)*, *lin(bh13)*, *lin(bh14)*, *lin(bh20)*, *lin(bh23)*, *lin(bh25)*, *lin(bh26)*, *lin(sy5197)*, *lin(sy5212)*, *lin(sy5336)*, *lin(sy5368)*, *lin(sy5425)*, *lin(sy5426)*, *unc(sy5505)*, *unc(sy5506)*.
Transgenic strains: *bhEx31[pRH51(hs*::*lin-3) + myo-2*::*GFP]*, *bhEx78[pGF50(lin-11) + myo-2*::*GFP]*, *bhEx117[mec-7*::*GFP + myo-2*::*GFP]*, *bhEx123[C07H6 + myo-2*::*GFP]*, *bhEx124[C07H6 + myo-2*::*GFP]*, *bhEx132[F44F12 + myo-2*::*GFP]*, *bhEx134[F44F12 + myo-2*::*GFP]*, *bhEx139[pSL38(unc-84) + myo-2*::*GFP]*, *bhEx141[pSL38(unc-84) + myo-2*::*GFP]*, *bhEx142[pSL38(unc-84) + myo-2*::*GFP]*, *bhEx148[pGF50(lin-11) + myo-2*::*GFP]*, *bhEx152[pSL38(unc-84) + myo-2*::*GFP]*; *mfIs5[Cbr-egl-17*::*GFP + myo-2*::*GFP]*, *mfIs8[Cbr-zmp-1*::*GFP + myo-2*::*GFP]*.

*mfIs5* and *mfIs8* animals carry a *gfp* reporter driven by the vulva-specific enhancers of *Cbr-egl-17* (748 bp) and *Cbr-zmp-1* (755 bp), respectively (Kirouac and Sternberg 2003). *bhEx117* is a transgenic HK104 line that was used in polymorphism-based mapping experiments (see below).

### Mutagenesis

AF16 animals were mutagenized by soaking in 25 mM ethyl methane sulfonate (EMS) and screening for Egl and Pvl mutants in the F2 generation. To prevent worms from burrowing into the agar, we used 9-cm NG-Agarose plates (1 L of media containing 3 g of sodium chloride, 2.5 g of bacteriological peptone, and 17 g of agarose; the other components were the same as nematode growth medium). Mutagenized worms were individually transferred onto plates, and the F2 progeny were screened for Egl worms. Such animals formed the characteristic “bag of worms” phenotype as a result of the progeny hatching inside the uterus and devouring the mother (Horvitz and Sulston 1980).

From four independent F2 screens (in the range of 100,000-125,000 haploid genome sets in total), we recovered 39 independent Egl clones that bred true. An additional 34 Egl clones could not be propagated because they were either sterile or gave rise to dead progeny. Apart from animals with the Egl phenotype, we also recovered dumpy and uncoordinated mutants. One of these, twitcher, was isolated from at least three independent plates (B. P. Gupta, unpublished results). In *C. elegans* and *C. briggsae*, the twitcher phenotype is associated with *unc-22*, a gene with more than 20 kb of open reading frame that is readily mutated in EMS screens (Benian *et al.* 1993). All three twitcher mutations are recessive and have been found to be allelic (data not shown), which suggests that our screens were capable of recovering viable recessive mutations with a visible phenotype.

This study focuses on a collection of 19 mutations that reside in 13 genes (see *Results*). Compared with the original *C. elegans* Egl screen (Trent *et al.* 1983), the number of Egl mutants in our case is considerably lower. It is unclear whether this is due to differences in the population of screened worms, as Trent *et al.* did not provide an estimate of the number of worms that were screened. Based on the mapping and complementation experiments, 70% of *C. briggsae* genes (9 of 13) are represented by single mutations (see *Results*). Although this result is indicative of the screen being unsaturated, the proportion of genes defined by a single allele in our case is very similar to that of Trent *et al.* (Trent *et al.* 1983). Furthermore, it is worth pointing out that additional alleles of the existing *C. briggsae* genes may be present among the remaining 20 mutations that have yet to be characterized. This analysis is the focus of our current study.

Similar to *C. elegans* (Ferguson and Horvitz 1985; Trent *et al.* 1983), not all *C. briggsae* mutants described here affect vulva formation, indicating that defects in other egg-laying components (such as neurons and muscles) can also lead to the Egl phenotype. Each mutant was backcrossed at least three times before we performed genetic experiments. All alleles were recessive and caused no obvious maternal effect phenotype.

### Microscopy, cell ablations, and VPC fates

Worms were mounted on agar pads as described previously (Wood 1988) and examined under Nomarski optics using Zeiss Axioimager D1 and Nikon Eclipse 80i microscopes. Sodium azide (1 M) was used as an anesthetic. To examine vulval lineages, L3 and L4 stage animals were mounted without any anesthetic, and coverslip edges were sealed with Vaseline to prevent dehydration. For GFP reporter-expressing strains, epifluorescence was visualized with a Zeiss Axioplan microscope equipped with the GFP filter HQ485LP (Chroma Technology), a power source (Optiquip 1500) and a 200 W OSRAM Mercury bulb. Cell ablation experiments were performed as described (Avery and Horvitz 1987).

VPC fates were examined in L3 and L4 stage animals under a Nomarski microscope. If a VPC fused with hyp7 as a single cell without dividing, it was assigned an ‘F’ (Fused) fate. If the VPC divided once and its daughters (Pn.px, where x denotes both anterior and posterior cells) fused with hyp7, it was assigned a 3° (tertiary) fate. If the VPC was induced to give rise to more than 4 vulval progeny (Pn.pxxx cells), it was considered fully induced and assigned an ’I’ (induced) fate [includes 1° and 2° fates as described previously (Sternberg and Horvitz 1986)]. Vulval induction score was calculated as described previously (Gupta *et al.* 2006). In *sy5353* and *sy5353*; *bh20* mutants some of the Pn.p appeared small and morphologically similar to P12.pa (Seetharaman *et al.* 2010). These were termed as “small” cells.

To determine inter-VPC distances in *lin-39* mutants, animals were bleach synchronized. Distances among the 5 VPC pairs (P3.p-P4.p, P4.p-P5.p, P5.p-P6.p, P6.p-P7.p, P7.p-P8.p) were measured in mid-to-late L2 stage animals using Nikon NIS Elements software.

### Pharmacological assays

Serotonin and fluoxetine were used to analyze the pharmacological response of some of the Egl mutants. Serotonin (35 mM) and fluoxetine (1 mg/mL) solutions were freshly prepared in M9 buffer. The assay was performed in 96-well microtiter dishes using 50 µL of drug in individual wells. As a control, the same volume of M9 buffer was placed in adjacent wells. L4 animals were picked a day before the assay and allowed to grow for 18-24 hr before placing them individually into drug and M9 containing wells. After incubating worms for 1 hr at room temperature, the number of eggs laid by each worm was counted. Assays were repeated at least three times.

### Heat shock protocol

L1 animals of the *bhEx31* strain were transferred to standard NG agar plates containing *Escherichia coli* OP50 bacteria and grown for a desired period of time. Plates were sealed with Parafilm M (American National Can) and heat shocked in a water bath. We tested various heat shock conditions by fixing the temperature at 37° and varying the duration of the exposure. Two different types of pulses, *i.e.* a single long pulse (between 0:30 hr and 1:30 hr) and multiple short pulses (either consisting of four 30-min pulses each separated by 1-hr rest period or two 1-hr pulses separated by 1h, *i.e*., 1-hr-r rest period), were tested. Animals were heat shocked at different time points after transferring L1 worms on bacteria-containing plates. After the initial trials, we chose 37° for 1 hr for all subsequent experiments. After heat shock treatment, animals were shifted back to 20°. Vulval induction and morphology were examined at stage L4.

### Egl penetrance assay

L4 animals were placed individually into six-well nematode growth medium–agar plates and observed over a 3-day period. Egl phenotype was classified as Egl (no laid eggs, “bag of worms” appearance), semi-egl (few eggs initially but eventually formed “bag of worms”), and Non-Egl (no defect, phenotypically wild type).

### Complementation tests

Complementation tests between two vulval mutants (*m1* and *m2*) were performed by crossing *m1*/+ heterozygote males (obtained by crossing *m1/m1* hermaphrodites to *myo-2*::*gfp* carrying *mfIs5* or *mfIs8* males) to *m2/m2* hermaphrodites. The presence of the *gfp* transgene allowed us to identify cross progeny. In the F2 generation, vulval phenotype in L4 worms was scored under Nomarski optics. Complementation tests were carried out for mutations belonging to the same phenotypic categories. Table 1 lists all combinations that were tested and the results.

**Table 1:** Results of complementation experiments

*m1/+*	*m2/m2*	Animals Showing Phenotype	Phenotype Scored
*bh2/+*	*bh6*	39% (n = 28)	Egl
*bh7/+*	*bh14*	0% (n = 60)	Vul
*bh7/+*	*bh20*	0% (n = 23)	Vul
*bh14/+*	*bh20*	0% (n = 31)	Vul
*sy5197 /+*	*bh13*	62% (n = 8)	Sma, vulval invagination abnormal
*bh13/+*	*bh25*	0% (n = 30)	Egl, Sma, vulval invagination abnormal
*sy5336/+*	*sy5368*	58% (n = 12)	Egl, vulval invagination abnormal

### Phenotypic marker-based genetic mapping

We tested the linkage of *lin-11(sy5336)* with several phenotypic markers that were assigned to various chromosomes. The website www.briggsae.org shows a larger list of mapping experiments involving these markers. The *sy5336* mutation was linked to *sma(sy5330)* (Table 2). Together these two genes define a single linkage group that was assigned chromosome 1 based on *sy5336* molecular identity and synteny of the *lin-11* genomic region (http://www.wormbase.org). The *unc(sy5506)* mutation was linked to chromosome X based on the Unc phenotype of F1 males derived from a cross of *sy5506* hermaphrodites to AF16 males.

**Table 2:** Linkage mapping of *Cbr-lin-11* using phenotypic markers

Marker	LG	Data
*sma(sy5330)*	*I*	2/39 Sma were Egl
*dpy(sy5148)*	*II*	19/29 Egl segregated Dpy
*dpy(5022)*	*III*	11/18 Egl segregated Dpy
*unc(s1270)*	*IV*	16/24 Egl segregated Unc
*unc(sa997)*	*V*	24/32 Unc segregated Egl
*unc(sy5077)*	*X*	15/32 Unc segregated Egl

### Insertion-deletion (indel) and snip-SNP-based genetic mapping

All mutations except *lin(bh14)* and *Cbr-lin-11* alleles were mapped to chromosomes by bulk segregant analysis (BSA) using Indels and snip-SNPs (Table 3, Supporting Information, Figure S1). The cross scheme was as follows. Hermaphrodites of a given mutant strain were crossed with either normal or GFP fluorescing *(bhEx117)* HK104 males. F1 cross progeny were picked and cloned. In the next generation (F2), phenotypically mutant and wild-type animals (20 each) were picked separately and processed to obtain genomic DNA. Genomic DNA was prepared by placing worms into 5 to 10 µL of lysis buffer (containing Proteinase K). The solution was incubated at 60° for 1 hr followed by heat inactivation of Proteinase K at 95°. This crude genomic DNA prep was frozen at –20° and used as a template in polymerase chain reaction experiments. The detailed indel mapping protocol and primers have been published previously (Koboldt *et al.* 2010). We reported earlier the single recombinant analysis of *lin(sy5506)* using the indel bhP26. The distance between the two loci was determined to be 10% (Koboldt *et al.* 2010).

**Table 3:** Linkage mapping of mutations by BSA and SNP-chip techniques

		Chromosomal Location	
Gene	Allele	BSA-Based	SNP Chip-Based
*egl(sy5395)*	*sy5395*	1: left arm (bhP19)	−
*lin(bh7)*	*bh7*	1 (cb-m142, cb650)	1: 4.5 Mb
*lin(bh13)*	*bh13*	1: left arm (bhP42)	1: 4 Mb
*lin(bh25)*	*bh25*	1*^a^* (cb650)	−
*egl(bh2)*	*bh2*	1: center (bhP42)	1: 7.5 Mb
*Cbr-lin-11*	*sy5336*	−	1: 7.9 Mb*^b^*
*Cbr-lin-39*	*bh20*	3: right arm*^c^* (bhP40)	−
*unc(sy5505)*	*sy5505*	5: center/right arm (bhP5, cb-m103)	5: 8.5 Mb
*lin(sy5425)*	*sy5425*	5: center (bhP5)	−
*Cbr-unc-84*	*sy5506*	X: right arm*^c^* (bhP26)	−
*egl(bh21)*	*bh21*	X (bhP25)	X: 11.5 Mb
*lin(bh14)*	*bh14*	I (bhP1)	−
*lin(bh26)*	*bh26*	X: right arm (bhP26)	X: 12.5 Mb

Tightly linked indel and snip-SNP markers are shown in brackets. Dashes (−) indicate a lack of map information.

a Likely to be located on the left arm.

b Previous study (Zhao *et al.* 2010).

c Previous study (Koboldt *et al.* 2010).

### SNP chip‒based genetic mapping

In addition to the aforementioned polymorphism-based BSA mapping, we used a microarray chip mapping approach to localize the mutations on chromosomes (Table 3). For this, a 12x oligo microarray chip containing approximately 4500 SNPs was designed using methods similar to those for *C. elegans* (Flibotte *et al.* 2009). An earlier version of the *C. briggsae* chip contained almost 9700 SNPs and was successfully used to map mutations (Zhao *et al.* 2010). *C. briggsae* Egl animals were mated with HK104 males, and F1 heterozygotes were cloned. In the F2 generation, 100 mutant worms were picked and allowed to grow on 10 6-cm Petri plates close to starvation. The worms were washed off with M9 buffer. Genomic DNA was extracted using the QIAGEN Blood and Tissue DNeasy kit (cat. no. 69504). DNA hybridization, measurement of fluorescence intensity and ratio analysis were performed as described previously (Flibotte *et al.* 2009; Maydan *et al.* 2007). Based on the mapping signal intensity and the arc of the signal (Figure S2), the approximate chromosomal locations of mutations were determined (Zhao *et al.* 2010). In some cases, such as *sy5505*, arc pattern was not obvious, rendering the analysis less reliable. Overall, the SNP-chip data agreed with seven of the indel and snip-SNP BSA mapping results (Table 3). Independent verification of these results by phenotypic-marker-based classical mapping has not been performed.

### Molecular biology and transgenics

Transgenic worms were generated by injecting DNA into the syncytial gonad of adult hermaphrodites using *myo-2*::*GFP* (pPD118.33) as a transformation marker (S. Q. Xu, B. Kelly, B. Harfe, M. Montgomery, J. Ahnn, S. Getz, and A. Fire, personal communication). The microinjection technique was described previously (Mello *et al.* 1991).

The pSL38 plasmid, which contained a *C. elegans unc-84* rescuing fragment (McGee *et al.* 2006), was injected at 4 ng/µL in *unc(sy5505)* and *unc(sy5506)* animals. Stable lines (*sy5505*: *bhEx141* and *bhEx142*, *sy5506*: *bhEx139* and *bhEx152*) were analyzed for the rescue of Unc, P cell migration, and Egl phenotypes.

The hs::*lin-3* transgenic animals, *bhEx31*, carry the pRH51 plasmid [50 ng/µL (Katz *et al.* 1995)]. pRH51 contains the EGF domain of *lin-3* along with a synthetic signal peptide. The expression of *lin-3* is under the control of the *hsp16-41* promoter (pPD49.83).

For the rescue of *Cbr-lin-39* mutants, *C. elegans* cosmids C07H6 and F44F12, containing the entire *lin-39* genomic region, were injected into *bh20* animals. Two stable lines were obtained for each cosmid (*bhEx123* and *bhEx124* with C07H6 at 20 ng/µL; *bhEx132* and *bhEx134* with F44F12 at 0.7 ng/µL). VPC induction and Egl phenotypes were analyzed in transgenic animals. A greater proportion of F44F12 stable lines showed rescue of the Egl phenotype compared to C07H6. Therefore, we focused on *bhEx132* and *bhEx134* transgenic animals for all subsequent analyses.

*Cbr-lin-11* cDNA was amplified using the ProtoScript first strand kit (NEB, #E6500S). The primers cb-lin-11-up-1 and cb-lin-11-down-2 (Table S1) were used. Whole RNA was prepared from the mixed stage animals using a previously described TRIZOL method (Burdine and Stern 1996). The *C. elegans lin-11*-rescuing plasmid pGF50 (Freyd 1991) was injected at 20 ng/µL. pGF50 contains a 19-kb subclone of cosmid ZK273 that was previously shown to rescue *C. elegans lin-11* mutants (Freyd 1991). Two stable lines (*bhEx78* and *bhEx148*) were generated for pGF50 (20 ng/µL), both of which rescued Egl and vulval invagination defects in *sy5336* animals.

### Sequencing

All primer sequences are listed in Table S1. The exons of *Cbr-unc-84* were amplified using primer pairs GL793/GL795, GL800/GL801, GL806/812, and GL809/810. To sequence the intermediate regions, primers GL802, GL807, and GL808 were used. A 403-bp deletion between exons 6 and 7 (genomic location +5006 and +5408) was identified that introduces an in-frame stop codon downstream of the deleted region.

*Cbr-lin-39* exons were amplified from *bh20* and *bh23* alleles using primer pairs GL380/GL381, GL382/GL383, GL384/GL385, GL389/GL390, and GL391/GL392. The *bh23* mutation contains a 364-bp deletion overlapping with the 5′ region of the *Cbr-lin-39* coding sequence. The deletion is located between -158 (upstream of the ATG start site) and +207 (in exon 1). The *bh20* allele carries a point mutation in exon 3 (G9427 to A) that corresponds to the homeodomain region.

The *Cbr-lin-11* ORF was amplified in two fragments using primer pairs cb-lin-11-up-1/cb-lin-11-down-7 and cb-lin-11-up-5/cb-lin-11-down-1. Sequencing primers were cb-lin-11-up-1, cb-lin-11-up-4, cb-lin-11-up-6, cb-lin-11-up-7, cb-lin-11-up-8, cb-lin-11-up-9, cb-lin-11-down-1, cb-lin-11-down-5, cb-lin-11-down-7, and cb-lin-11-down-8. Both *lin-11* alleles, *sy5336* and *sy5368*, affect splicing. *sy5336* causes a G to A transition (G4403 to A) in the splicing acceptor site of intron 7 and is likely to disrupt intron 7 splicing. The *sy5368* mutation affects the splicing donor site of intron 6 (G3340 to A) and is predicted to introduce a premature in-frame stop codon 52 nucleotides downstream.

### Statistical analysis

Statistical analyses were performed using InStat 2.0 (GraphPad) Software. Two-tailed *P* values were calculated in unpaired *t*-tests, and values less than 0.05 were considered statistically significant.

## Results

### Overview of the genetic screen

We screened for egg-laying defective (Egl) mutants after EMS mutagenesis of AF16 animals (see *Materials and Methods* for details). Of 39 Egl mutants identified, we report the characterization of 19 mutants. Seventeen of these fell into 13 complementation groups and were placed into four distinct phenotypic categories (Table 4). Of the remaining 2, *lin(sy5212)* and *lin(sy5426)*, *sy5212* is a fully penetrant Vul mutant and could not be outcrossed. In rare circumstances, VPC induction in *sy5212* animals was observed only for P6.p. All other VPCs fused to hyp7 during the L2 and L3 stages. The other mutant, *sy5426*, has variable vulva defects (a combination of missing VPCs, uninduced VPCs, and abnormal morphogenesis) and could not be uniquely classified. We used indel-based BSA, snip-SNP, and SNP-chip mapping approaches (Koboldt *et al.* 2010; Zhao *et al.* 2010) to localize the mutations to chromosomes (Table 3).

**Table 4:** Overview of *C. briggsae* egg-laying defective mutants

					Egl Penetrance (%)			
Class	Features	Gene	Alleles	Mutation	Non-Egl	Semi-Egl	Egl	n
1	Wild-type vulva	*egl(bh2)**^a^*	2	*bh2*	0	69	31	103
		*egl(bh21)*	1	*bh21*	0	41	59	120
		*egl(sy5395)*	1	*sy5395*	6	32	62	244
2	Fewer Pn.p cells	*unc(sy5505)*	1	*sy5505*	22	17	61	127
		*Cbr-unc-84*	1	*sy5506*	56	21	23	100
3	Reduced VPC induction	*lin(bh7)*	1	*bh7*	81	12	7	137
		*lin(bh14)*	1	*bh14*	39	32	29	133
		*Cbr-lin-39*	2	*bh20*	0	4	96	140
				*bh23*	0	0	100	41
4	Abnormal vulval invagination	*Cbr-lin-11*	2	*sy5336*	0	0	100	100
				*sy5368*	0	0	100	100
		*lin(bh13)^#^*	2	*bh13*	1	1	98	102
		*lin(bh26)*	1	*bh26*	0	0	100	100
		*lin(bh25)*	1	*bh25*	1	23	76	105
		*lin(sy5425)*	1	*sy5425*	36	25	39	104
−	Unclassified	*lin(sy5212)*	−	*sy5212*	0	0	100	49
		*lin(sy5426)*	−	*sy5426*	0	0	100	29

Egl, animals did not lay eggs at all; n: number of animals scored; Non-Egl, animals continued to lay eggs throughout their reproductive life; Semi-Egl, animals laid eggs initially but became Egl afterward.

a The phenotype of the other allele of this locus was not characterized in detail.

Class 1 mutants consist of three loci, each of which shows morphologically wild-type vulval development and a vulva-uterine connection (utse). The Egl phenotype of these animals is likely to result from defects in neuronal and/or muscle components of the egg-laying system. Class 2 is composed of two mutants, both of which are uncoordinated and frequently lack VPCs. In some cases, these animals lack a functional vulva and develop an Egl phenotype. Class 3 mutants are represented by three loci, each of which shows reduced VPC induction. The strongest allele in this class, *bh23*, causes a fully penetrant Egl defect. The largest phenotypic category, class 4, is composed of five loci. Mutations belonging to this class do not affect VPC induction but cause abnormal vulval invagination and morphology. The adults frequently have Pvl and Egl phenotypes.

### Class 1 mutants have defects in egg-laying components other than the vulva and utse

**Figure 1:**
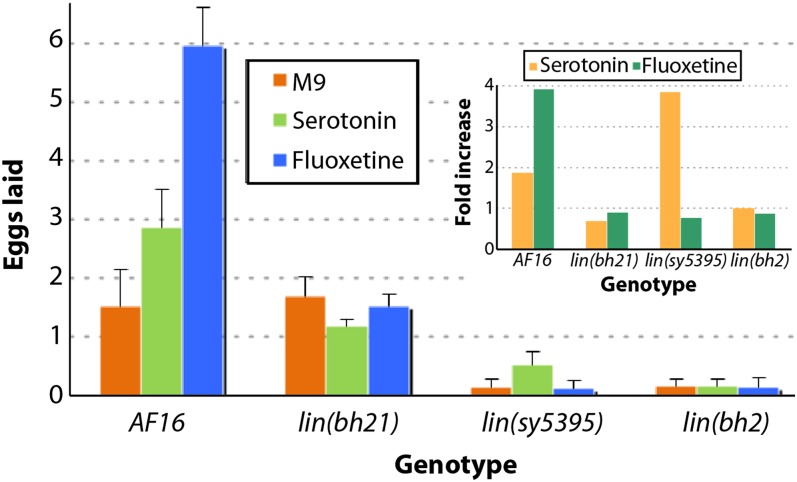
Egg-laying responses of class 1 mutants. The graph shows the average number of eggs laid by animals in M9 buffer (control) and serotonin and fluoxetine drug–containing solutions. The fold increase in egg laying in drug solution (over M9 buffer control) is plotted in the inset graph.

The examination of vulval phenotype in class 1 mutants revealed that VPCs and their progeny were unaffected. Vulval cells invaginated correctly and gave rise to a morphology characteristic of the wild-type animals. Furthermore, the utse was normal and was located on the top of the vulval apex (data not shown). To examine defects in other components of the egg-laying system, we treated animals with drugs that affect neuronal and muscle activities. In *C. elegans*, hermaphrodite-specific neurons (HSNs) control egg-laying behavior (Croll 1975; Horvitz *et al.* 1982; Weinshenker *et al.* 1995). In response to external cues, such as food, HSNs release serotonin (*i.e.*, 5-hydroxytryptamine or 5-HT) into the neuromuscular synapse, which then acts on the postsynaptic receptors in the vulval muscle to stimulate the release of eggs. Authors investigating the role of HSNs have used serotonin and fluoxetine [a serotonin reuptake inhibitor that increases the amount of neurotransmitter available to post-synaptic receptors (Baldessarini 1996; Dempsey *et al.* 2005)] to characterize the neuronal basis of the Egl phenotype. Serotonin and fluoxetine drug assays can distinguish between pre- and postsynaptic defects (*i.e.*, between HSN and vulva muscle). Mutants resistant to fluoxetine that lay eggs in the presence of exogenous serotonin are likely to have abnormal HSNs, whereas resistance to both drugs suggests a postsynaptic signaling defect. We found that *egl(sy5395)* animals, when exposed to serotonin, had a modest but consistent increase in the number of eggs laid compared with the control, but *egl(bh21)* and *egl(bh2)* were unaffected (Figure 1). Fluoxetine exposure had no obvious effect on any of the strains. These results suggest that the Egl phenotype in *sy5395* animals may be caused by abnormal differentiation of HSNs. In the case of *bh21* and *bh2* mutants, the cellular basis of the Egl phenotype remains to be identified.

### Class 2 mutants have defects in nuclear migration and include *Cbr-unc-84*

**Figure 2:**
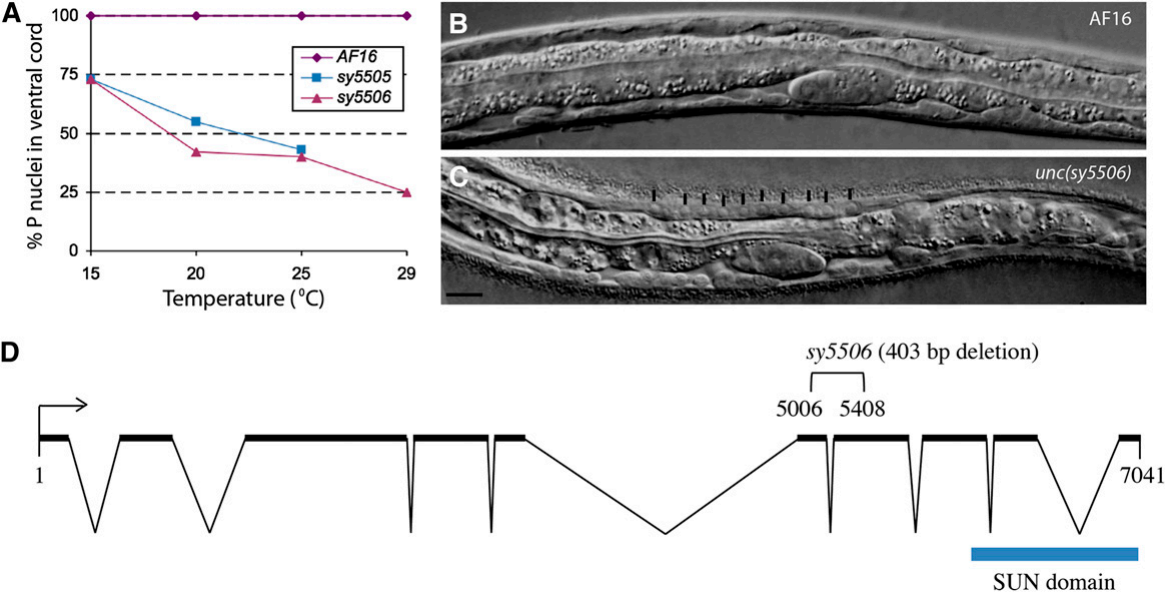
Nuclear migration defects in class 2 mutants and molecular analysis of *Cbr-unc-84*. (A) P-cell nuclei in the ventral cord region of mutants vary with temperature. At greater temperatures, fewer nuclei are visible. *sy5505* animals are very sick at 29° and could not be examined. Each data point consists of 25 or more animals. (B, C) Wild-type AF16 and *sy5506* L1 stage animals, respectively. The hyp7 nuclei in the *sy5506* animals fail to migrate and are located in the dorsal region (marked with vertical lines). Scale bar is 10 µm. (D) Open reading frame of *Cbr-unc-84*. *sy5506* causes a 403-bp deletion.

**Figure 3:**
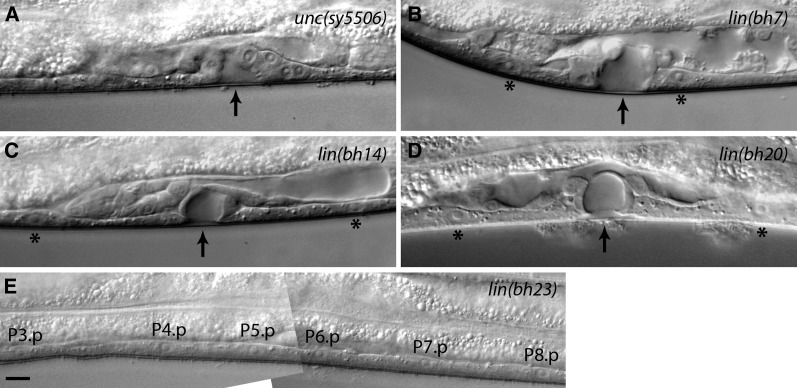
Vulva phenotypes of class 2 and 3 mutants. Arrows point to the center of invagination. Animals were examined at the mid-L4 stage. (A) Fewer VPCs are present in *sy5506* animals due to a defect in P-cell nuclear migration. In this case P6.p and P7.p are induced to form the vulva. P5.p and P8.p are missing. (B) One or more VPCs in *bh7* animals remain uninduced. In this example, P5.p and P6.p are induced. P4.p and P7.p have adopted an F fate (asterisks). (C, D) *bh14* and *bh20* animals showing a similar vulval morphology defect. In both cases invagination is formed by P6.p progeny, whereas P5.p and P7.p have adopted an F fate (asterisks). (E) A *bh23* animal showing no vulval induction. All VPCs have adopted an F fate. Scale bar is 10 µm.

The class 2 mutants *unc(sy5505)* and *unc(sy5506)* have fewer and more variable numbers of P cells in the ventral hypodermal region. Animals homozygous for either of these mutations move in a slow and uncoordinated manner. Microscopic observations revealed fewer than 12 P-cell nuclei in the ventral hypodermal region (Figure 2A). This phenotype was temperature sensitive, such that the loss of P cells was greater at higher temperatures (Figure 2A and data not shown). Nearly two-thirds of the animals had an Egl phenotype due to the absence of some or all of the P(5-7).p VPCs (Tables 4 and 5, Figure 3A). We also observed a hyp7 nuclear migration defect in the *sy5506* strain. Unlike wild-type animals where no hyp7 nuclei are observed in the dorsal hypodermis, *sy5506* worms had many hyp7 nuclei in this region (Figure 2, B and C).

**Table 5:** Vulval induction pattern in Class 2 and 3 mutants

			% VPC Fate Pattern (F/3°/I)						
Class	Genotype	VPC Induction Score	P3.p	P4.p	P5.p	P6.p	P7.p	P8.p	n
	AF16	3	61/39/0	0/100/0	0/0/100	0/0/100	0/0/100	0/100/0	101
2	*unc(sy5505)*	1.7 ± 1.3	39/4/0	0/35/0	0/0/50	0/0/75	0/0/48	2/37/0	52
	*sy5505*; *bhEx141*	1.2 ± 1.2	25/2/00	8/8/00	0/0/31	0/0/62	0/0/31	4/13/0	52
	*sy5505*; *bhEx142*	1.4 ± 1.1	24/4/0	2/18/0	0/0/38	0/0/62	0/0/38	4/11/0	45
	*Cbr-unc-84(sy5506)*	1.9 ± 1.2	31/14/0	10/18/0	0/0/53	0/0/82	0/0/51	14/25/0	51
	*sy5506*; *bhEx139*	2.9 ± 0.4*^a^*	54/30/0	0/86/0	0/0/96	0/0/100	0/0/94	0/93/0	91
	*sy5506*; *bhEx152*	2.9 ± 0.4*^a^*	57/31/0	2/90/0	0/0/94	0/0/100	0/0/96	0/94/0	51
3	*lin(bh7)*	2.3 ± 1	77/23/0	54/46/0	28/4/68	5/0/95	32/2/66	43/57/0	56
	*lin(bh14)*	2.3 ± 1	91/9/0	71/29/0	35/0/65	1/0/99	33/0/67	65/35/0	78
	*Cbr-lin-39(bh20)*	0.2 ± 0.4	100/0/0	100/0/0	100/0/0	85/0/15	100/0/0	100/0/0	155
	*bh20*; *bhEx134*	1 ± 0.9*^a^*	100/0/0	97/3/0	93/0/7	40/0/60	71/0/29	99/1/0	88
	*bh20*; *bhEx132*	0.5 ± 0.5*^a^*	100/0/0	100/0/0	99/0/1	54/0/46	100/0/0	100/0/0	81
	*Cbr-lin-39(bh23)*	0	96/4/0	96/4/0	88/12/0	96/4/0	96/4/0	100/0/0	50

VPC fates are classified into three categories: F, fused with hyp7 without division, 3°, fused with hyp7 after one cell division, I, induced (either 1°, 2°, or a hybrid fate that could not be uniquely classified). See *Materials and Methods* for details. For *sy5505* and *sy5506* animals missing VPCs were excluded from the analysis. VPC, vulval precursor cells.

a VPC induction is significantly higher compared to the parental strain, *P* < 0.0001.

In *C. elegans*, similar phenotypes are caused by mutations in two genes, *unc-83* (KASH domain) and *unc-84* (SUN domain), which affect nuclear migration during development (Malone *et al.* 1999; Starr *et al.* 2001; Sulston and Horvitz 1981). One of these, *unc-84*, is located on the right arm of chromosome X. We found that *sy5506* also maps to the right arm of chromosome X close to the bhP27 polymorphism (see *Materials and Methods*), a region that contains *Cbr-unc-84*/*CBG07416* and is syntenic to *unc-84*.

To further confirm that *unc(sy5506)* defines the *Cbr-unc-84* gene, we generated transgenic *sy5506* animals carrying an *unc-84* rescuing plasmid called pSL38 (McGee *et al.* 2006). The transgenic animals showed rescue of the hyp7 and P-cell nuclear migration defects (51% *bhEx139* animals with normal hyp7 nuclear migration, n = 35, and 90% P nuclei present in ventral cord, n = 91, at 20°, compared with 100% abnormal hyp7 nuclear migration and 42% P-cell nuclei, n = 23, in *sy5506*). The Egl and VPC induction defects in mutants also were rescued (69% of *bhEx139* animals laying eggs, n = 94, compared with 56% in *sy5506*, n = 100; see Table 5 for VPC induction).

Finally, we sequenced the *Cbr-unc-84* genomic region in *unc(sy5506)* animals and identified a 403-bp deletion covering parts of exons 6 and 7 (Figure 2D, also see *Materials and Methods*). These results demonstrate that *sy5506* is an allele of *Cbr-unc-84*. In *C. elegans*, UNC-84 protein contains a SUN domain, a transmembrane domain and an intervening linker region (Malone *et al.* 1999; McGee *et al.* 2006). Based on the sequence alignment, the *sy5506* mutation is located in the linker region of Cbr-UNC-84. In *C. elegans*, this region interacts with UNC-83 to facilitate localization of other cytoskeletal components that are crucial for nuclear positioning in hyp7 and P cells.

The phenotype of *sy5505* animals differs from *Cbr-unc-84(sy5506)* in two respects. First, they are more sensitive to increased temperature as evidenced by their inability to grow at 29° (Figure 2A). Second, no hyp7 migration defect was observed in *sy5505* animals (data not shown). We also generated transgenic *sy5505* strains carrying the *unc-84* plasmid pSL38 (*bhEx141* and *bhEx142*) but did not observe rescue of the mutant phenotype (Table 5 and data not shown). These results together with linkage data (Table 3) suggest that *sy5505* is not allelic to *Cbr-unc-84* and is likely a different gene. The phenotype of *sy5505* is similar to mutations in *unc-83* in *C. elegans*; however, the possibility that *sy5505* is an allele of *Cbr-unc-83* has not been tested.

### Class 3 mutants exhibit reduced VPC induction

Four mutations define class 3 genes, all of which cause a reduction in the number of vulval progeny (see VPC induction score in Table 5) and abnormal invagination (Figure 3). In these animals, some or all P(3-8).p fail to divide and fuse with surrounding hypodermis (‘F’ fate; Table 5). The phenotype is weakest in *lin(bh7)* (only one VPC uninduced; Figure 3B) but fully penetrant in *lin(bh23)* (all VPCs uninduced; Figure 3E). The other two alleles, *lin(bh14)* and *lin(bh20)*, are intermediate (Figure 3, C and D), with *bh20* being somewhat more severe as determined by fewer cases of P6.p induction (15%; see Table 5) and rudimentary vulval invagination. This observation agrees well with the vulval induction score, cell lineage, and Egl penetrance of the animals (Tables 4, 5, and 6). The *bh20* and *bh23* mutations also cause abnormal folding of gonad arms and subtle uncoordinated phenotypes (data not shown).

**Table 6:** Vulval cell lineage analysis of class 3 and 4 mutants

VPCs						
Genotype	P3.p	P4.p	P5.p	P6.p	P7.p	P8.p	n
*AF16*	S/SS	SS	LLTN	TTTT	NTLL	SS	>50
*lin(bh7)*	SS	SS	LLTN	TTTT	NTLL	SS	4
	S	SS	LLTN	TTTT	NTLL	SS	1
	S	S	S	TTTT	NTLL	SS	1
	S	SS	LLTN	TTTT	NTLL	OOLL	1
	S	S	SS	TTTT	NTLL	SS	1
	S	SS	LLTN	TOOT	S	SS	1
	S	S	S	TTTT	S	S	1
*lin(bh14)*	S	S	S	TTTT	OTLL	S	1
	S	S	LLTN	TTTT	NTLL	S	2
	S	S	LLTN	TTTT	NTLL	SS	1
	S	S	S	TTTT	S	S	2
	S	SS	NTOL	TTTT	S	S	1
	S	S	S	OOTT	S	S	1
	S	S	S	TTTT	S	S	1
	S	S	LLTN	TTTT	S	S	2
*lin(bh20)*	S	S	S	S	S	S	13
	S	S	S	TTTT	S	S	3
	S	S	S	OOOO	S	S	1
	S	S	S	TTTD	S	S	1
	S	S	S	OTTT	S	S	1
*lin(bh23)*	S	S	S	S	S	S	12
*lin(sy5336)*	SS	SS	LLLL	LTTT	LLLL	SS	2
	SS	SS	LLLL	ODTO	LLLL	SS	1
	S	SS	LLLL	OOOO	LLLL	SS	1
	SS	SS	LLLL	OOTO	LLLL	SS	1
	S	SS	LLLL	OTOT	LLLL	SS	1

Cells attached to the cuticle are underlined. D, division plane not observed; L, longitudinal; O, oblique; N, no cell division; n, number of animals scored; S, cell fused with syncytium; T, transverse plane of cell division.

Next, we examined the VPC induction defect in some detail. As the defect in *bh7*, *bh14*, and *bh20* animals is predominantly limited to 2° precursors (P5.p and P7.p), we wanted to determine whether these two VPCs lack the potential to respond to an external signal and are unable to adopt an induced fate. In *C. elegans*, AC is necessary for VPC induction because it secretes LIN-3/EGF ligand that activates the LET-23/EGFR-LET-60/RAS-MPK-1/MAPK pathway in VPCs (Hill and Sternberg 1992; Kimble 1981). To this end, we ablated the central VPC, *i.e.*, P6.p, during the L2 stage and examined the fates of the remaining VPCs. We predicted that P5.p and P7.p would receive greater levels of AC signal, perhaps triggering VPC induction. In wild-type animals, P6.p ablation causes full induction of P5.p and P7.p, whereas P4.p and P8.p adopt vulval fates in some cases (Table 7). In our experiment, *bh7* animals exhibited an induction pattern similar to AF16. Thus, P5.p and P7.p were induced in all cases (Table 7 compared with intact *bh7* animals in Table 5). The frequencies of induced VPCs were much lower in *bh14* animals (Table 7). In total, five of nine animals had some vulval tissue as a result of P5.p and P7.p induction (P5.p adopted 1° fate in three cases and P7.p in the remaining two). Only one of these animals had induction of both P5.p and P7.p (P5.p 1° and P7.p 2°). The remaining VPCs adopted an ‘F’ fate. Similar manipulations in *bh20* animals also caused P5.p and P7.p to be induced, albeit rarely (Table 7). Of the 19 cases, 4 had few vulval progeny. In two of these, P5.p appeared to adopt a 1°–like fate (no P7.p induction), whereas the other two had a hybrid 1°/2° lineage. This result is in contrast to intact *bh20* animals in which P5.p and P7.p are never induced (Table 5). Taken together, these results suggest that in the absence of the central P6.p, the neighboring VPCs in these three mutants can be induced by the AC-mediated signal, and they can give rise to vulval tissue.

**Table 7:** Effect of cell ablations on VPC fates in class 3 mutants

	VPC Fate (Induced/Uninduced)						
Genotype	P3.p	P4.p	P5.p	P6.p	P7.p	P8.p	n
*mfIs5(egl-17*::*gfp)*	0/100	30/70	100/0	x	100/0	70/30	10
*lin(bh7)*	0/100	67/33	100/0	x	100/0	17/83	6
*lin(bh14)*	0/100	0/100	33/67	x	22/78	0/100	9
	0/100	0/100	x	x	100/0	0/100	1
*lin(bh20)*	0/100	0/100	15/85	x	5/95	0/100	19

‘x’ denotes VPCs that were ablated during the early-L2 stage. See Table 5 for a description of VPC fates. Uninduced refers to F and 3° fates. n, number of animals scored; VPC, vulval precursor cells.

### Overexpression of *lin-3* suppresses the VPC induction defect in a subset of class 3 mutants

**Figure 4:**
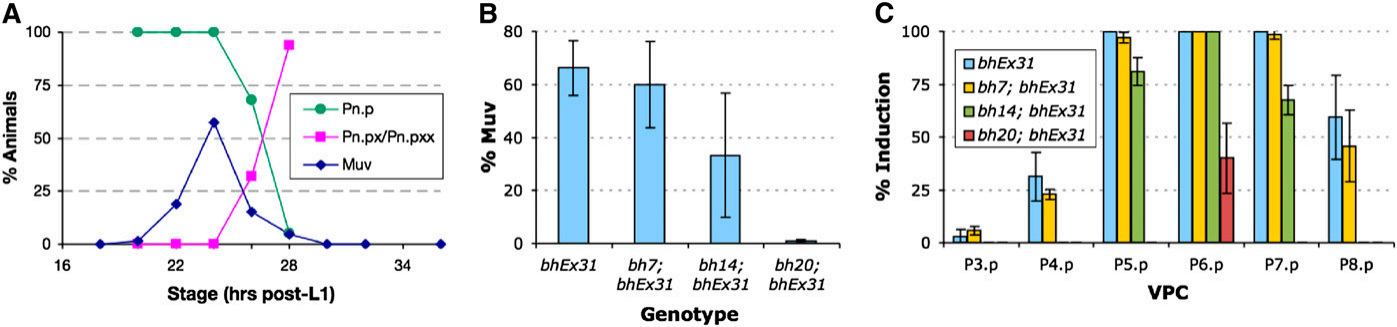
Effect of *lin-3* overexpression on VPC induction in class 3 mutants. The *bhEx31* transgenic strain carries a *hs*::*lin-3* plasmid from *C. elegans* (see *Materials and Methods* for details). (A) The graph shows the proportion of Pn.p (1-cell stage), Pn.px (2-cell stage), and Pn.pxx (4-cell stage) *bhEx31* animals at different time points of development (green and pink lines). The heat shock-induced Muv phenotype of *bhEx31* is also plotted (blue line). Almost 60% of animals, when subjected to heat shock at 24 hr after L1, develop a Muv phenotype. The Muv penetrance decreases rapidly after the VPCs start to divide such that by 30 hr, when all VPCs have divided, heat shock has no effect on VPC induction (no Muv phenotype develops). Pn.p and progeny stages were determined from a total of 16 to 20 animals for each time point. For Muv penetrance analysis, each time point contained (starting from the L1+18 hr stage) 25, 63, 328, 118, 300, 214, 86, 25, and 81 animals, respectively. (B) The graph shows the Muv phenotype in mutants after the heat shock at 24 hr. The Muv frequency in *bh7* animals is similar to the control *(bhEx31)*, slightly reduced in *bh14*, and not present in *bh20* animals. The number of animals examined in each case was 154 (control), 50 (*bh7*), 159 (*bh14*), and 112 (*bh20*). (C) The pattern of VPC induction in animals plotted in graph B. Although all VPCs can be induced in the control and *bh7* to varying extents, only the central 3, P(5-7).p, do so in *bh14*, and only one (P6.p) is induced in *bh20* animals. For each genotype, we examined 32 (control), 70 (*bh7*), 37 (*bh14*), and 95 (*bh20*) animals.

In *C. elegans*, LIN-3 signaling plays a role in maintaining the competence of VPCs by preventing their fusion with hyp7 (Myers and Greenwald 2007). This allows unfused VPCs to initiate Ras-MAPK signaling to promote vulval induction. A similar mechanism could operate in *C. briggsae* as well, which would be consistent with the results of our aforementioned cell ablation experiments in which P5.p and P7.p often were induced in the absence of P6.p, possibly by responding to a greater level of gonad-derived signal. To test this directly, we monitored the effect of increased doses of LIN-3/EGF on VPC induction in *bh7*, *bh14*, and *bh20* animals. A *C. elegans lin-3* transgene under the control of a heat shock promoter was introduced in *C. briggsae*. This transgene was previously used in *C. elegans* and causes a Muv phenotype (Katz *et al.* 1995). Heat shocks given during early stages (0-18 hr post-L1) and late stages (30 hr post-L1 and beyond) had no effect on vulval development (Figure 4A and data not shown). However, 20-28 hr post-L1, animals (VPC one-cell stage, Pn.p) developed a Muv phenotype when subjected to heat shock (Figure 4A). The Muv penetrance was highest at the 24 hr post-L1 time point (58%, see Figure 4A, AC visible in all cases at the time of heat shock), which corresponds to the late-L2/early-L3 stage and precedes the division of dorsal uterine precursors. All VPCs were induced, although P3.p appeared to be somewhat refractory in this assay (Figure 4C).

Next, we examined the effect of *lin-3* overexpression in *bh7*, *bh14* and *bh20* animals. Heat shocks at the 24 hr post-L1 time point induced a Muv phenotype in *bh7* animals similar to the control (Figure 4B). The *bh14* animals showed a similar but reduced response (Figure 4B). In contrast, no Muv phenotype was observed in *bh20* animals (Figure 4B). Examination of cell fates revealed that greater doses of LIN-3 induced VPCs in all 3 genetic backgrounds, including *bh20* in which P6.p was threefold more likely to be induced compared to the control (Figure 4C). These results show that increased VPC induction in class 3 mutants is most likely caused by the activation of a pathway homologous to LIN-3/EGF signaling in *C. elegans*.

### Class 3 mutations *bh20* and *bh23* are alleles of *Cbr-lin-39*

**Figure 5:**
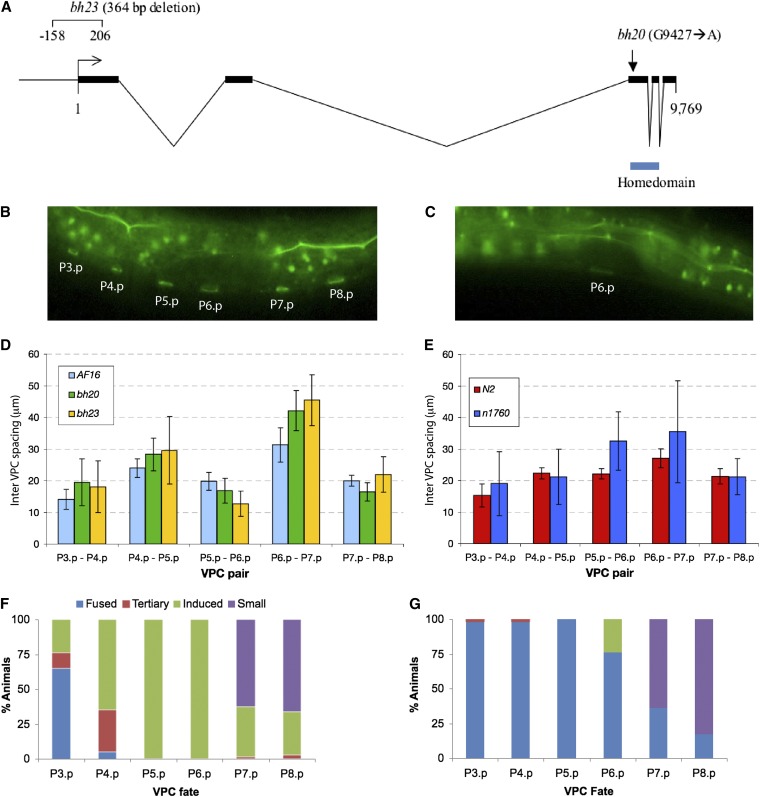
Genomic organization and mutant phenotypes of *lin-39*. (A) *C. briggsae lin-39* open reading frame. Exons are represented by thick lines. The positions of the homeodomain and two mutations are shown. (B, C) Unfused VPCs revealed by *dlg-1*::*GFP* expression. (B) Wild-type AF16 and (C) *bh20* L2 stage animals. In this *bh20* animal only P6.p ring is visible. (D, E) Inter-VPC spacing in *lin-39* alleles in *C. elegans* and *C. briggsae*. VPCs are irregularly spaced in mutants, which is reflected in greater SDs. (F, G) Excessive induction of VPC phenotype in *sy5353* animals is suppressed by *bh20*. (F) *sy5353* and (G) *bh20*; *sy5353* double mutant. VPC fates are shown as Fused, 3°, induced (1° and 2°) and small (P12.pa-like).

The cell fusion defect in class 3 mutants was similar to that of *C. elegans* with mutant Hox gene *lin-39* alleles that cause P(3-8).p cells to fuse to surrounding hypodermis (Clark *et al.* 1993). Therefore, we wanted to determine whether any of these are alleles of *Cbr-lin-39*. Using Indel mapping, we had earlier placed *lin(bh20)* on the right arm of chromosome 3 (Koboldt *et al.* 2010). This region includes *C. elegans lin-39* ortholog (http://www.wormbase.org). Therefore, we took a candidate gene approach and sequenced the exonic regions of *Cbr-lin-39* in *bh20* animals. A single point mutation (G9427 to A) was found that is predicted to replace a conserved arginine (R) with glutamine (Q) at position 169 in the homeodomain region (Figure 5A). Considering that *lin(bh23)* animals show a similar but more severe VPC induction defect, we suspected that this could be another allele of *Cbr-lin-39*. Sequencing of the *Cbr-lin-39* region in this strain identified a 364 bp deletion affecting the promoter and translational start site (Figure 5A).

We also carried out a transgene rescue experiment for *lin(bh20)* animals using a *C. elegans lin-39*-rescuing genomic DNA clone. Examination of transgenic animals revealed a rescue of the VPC competence defect in both cases (Table 5). The Egl defect also was rescued in these lines (animals laying eggs: *bhEx132* 83%, n = 29 and *bhEx134* 24%, n = 124; compared with 4% in *bh20* alone, n = 140).

### Pn.p cells lack competence and are irregularly placed in *Cbr-lin-39* mutants

In *C. elegans*, *lin-39* acts at multiple times during vulval development. In the L1 and L2 stages, it prevents Pn.p cells from fusing to hyp7 (Clark *et al.* 1993). Later on in stage L3, it is up-regulated by Ras signaling to promote vulval induction (Maloof and Kenyon 1998). To determine whether *Cbr-lin-39* mutants cause cell fusion defects in *C. briggsae*, we used a junction-associated marker *dlg-1*::*GFP* (Seetharaman *et al.* 2010). In wild-type AF16, P(3-8).p remain unfused in the late-L1 and L2 stages and become competent to form the vulval tissue (Figure 5B). In the majority of *bh20* animals, the corresponding cells were fused by early-to-mid-L2 stage (60.7%, n = 28). In the remaining cases, one or two Pn.p cells were protected (P5.p and P6.p 21.4% and P6.p alone 17.8%, n = 28; Figure 5C). The proportion of animals with unfused cells was much lower at later stages and was limited to P6.p (mid-L3 stage: P6.p 30.8%, n = 13; L4 stage: P6.p 12.5%, n = 24).

As it has been previously reported that the spacing of Pn.p cells in the ventral hypodermis is less regular in *C. elegans lin-39* mutants (Clark *et al.* 1993), we measured VPC spacing in *Cbr-lin-39* animals. Our results agreed with the findings in *C. elegans* and revealed that both *Cbr-lin-39* alleles cause greater variability in VPC placement (Figure 5, D and E). Although all inter-VPC distances were affected, the phenotype was most pronounced in the P6.p-P7.p pair. Interestingly, the P5.p-P6.p pair in *C. briggsae* showed the reverse of *C. elegans*.

### *bh20* is epistatic to *Cbr-pry-1(sy5353)*

We recently demonstrated a conserved role for *pry-1*−mediated Wnt signaling in 2° VPC fate specification (Seetharaman *et al.* 2010). In *Cbr-pry-1*, animals P5.p and P6.p are always induced, but P7.p often is not (Figure 5F). The inability of P7.p to contribute to vulval tissue is likely due to a change in cell fate as judged by its small nucleus and P12.pa-like appearance. *Cbr-pry-1* mutants also exhibit ectopic induction of P3.p and P4.p and to some extent P8.p, resulting in the formation of multiple ventral protrusions (pseudovulvae). Thus, both a lack of induction (P7.p) and excessive induction phenotypes are observed in *Cbr-pry-1* animals.

Given that *lin-39* acts genetically downstream of *pry-1* in *C. elegans* (Gleason *et al.* 2002), we examined the genetic interaction of *Cbr-lin-39* with *Cbr-pry-1*. As expected, *Cbr-lin-39*(*bh20*) suppressed the increased induction phenotype of *Cbr-pry-1(sy5353)*. Ectopic vulval induction was inhibited due to VPCs frequently adopting F fates (n = 42; P6.p was induced in 24% cases; Figure 5G). However, the small nucleus phenotype of P7.p, P8.p, and P11.p was not suppressed by *bh20* (Figure 5G and data not shown). Therefore, one of the following may be true: either the residual activity of *Cbr-lin-39* in *bh20* animals is greater than the threshold needed for VPC size specification or *Cbr-lin-39* mediates only a subset of *Cbr-pry-1* function.

### Class 4 mutations affect vulval invagination and morphology

**Figure 6:**
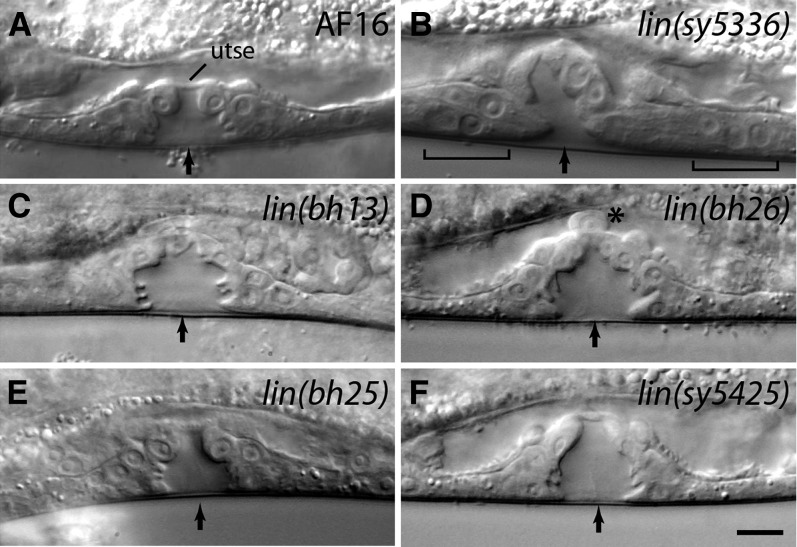
Vulval morphology defects in class 4 mutants. Animals were examined at mid-L4 stage. Arrows mark the center of invagination. (A) Wild type. The utse is visible as a thin line above the vulva. (B) The 2° lineage vulval cells fail to invaginate (shown by brackets). The utse cannot be clearly identified. (C, D) The vulval opening is blocked due to a failure in the migration of the 1° lineage cells. (E, F) Vulval invagination is abnormal. In addition, the utse in a *bh25* animal (E) is thicker compared with the wild type. Scale bar is 10 µm.

This class consists of seven mutants, all of which have defective vulval morphology (Table 8). Microscopic observations revealed that the animals have the correct number of VPCs and their progeny but the cells fail to invaginate correctly (Figure 6). In addition to the Egl phenotype, the adults exhibit a Pvl phenotype (Table 8). Complementation and mapping experiments revealed a total of five loci, three of which are located on chromosome 1 with the others on chromosome 5 and the X chromosome (Table 3). Two of the chromosome 1 genes have additional alleles (*bh13* and *sy5197* on the left arm, *sy5336* and *sy5368* close to the center).

**Table 8:** Vulval morphology defects in class 4 mutants

Gene	Allele	Abnormal Vulval Morphology	Pvl
*lin(bh13)*	*bh13*	71% (69)	29% (110)
	*sy5197*	51% (39)	ND
*lin(bh25)*	*bh25*	47% (36)	7.4% (148)
*lin(bh26)*	*bh26*	86% (51)	51.8% (110)
*lin(sy5425)*	*sy5425*	83% (35)	17.9% (95)
*lin(sy5336)*	*sy5336*	100% (100)	83.9% (152)
	*sy5368*	100% (100)	51.2% (162)

Number of animals scored are shown inside parentheses. ND, not done; Pvl, protruding vulva.

The *sy5336* and *sy5368* mutations cause a fully penetrant Egl phenotype. A distinctive feature of these animals, with regards to egg laying, is rudimentary vulval invagination and defects in the connection of the vulva to the uterus (Figure 6B). The analysis of VPC lineages revealed errors in cell divisions and adherence properties of some of the 2° lineage cells (Table 6). Other phenotypes included low brood size (*sy5336*: 21 ± 5, n = 11; *sy5368*: 10 ± 2, n = 12) and defective mating. The hermaphrodites are unable to mate at all, whereas males can mate, although very poorly (data not shown).

The *bh13* and *sy5197* animals are small and mildly sluggish. Some also have abnormally folded gonad arms. These phenotypes accompany Egl and vulval invagination defects in both outcrossed strains (Tables 4 and 8, Figure 6C), suggesting they are linked to a single gene. We looked at the vulval morphology in L4 stage animals and found that the 1° lineage cell nuclei are abnormally placed. In addition, the utse cannot be clearly observed in these animals (Figure 6C). A combination of the vulva and utse defects appears to cause a physical block in the egg-laying passage.

The vulval morphology defect in *bh26* animals shares some similarity with that of *bh13* animals (Figure 6D compared with Figure 6C). Specifically, the nuclei of the 1° lineage cells fail to migrate correctly, thereby blocking the connection between the vulva and the uterus. We also observed defects in the migration of the AC and a lack of utse (Figure 6D). Other defects included abnormal gonad arms and sterility.

The remaining two class 4 mutants, *lin(bh25)* and *lin(sy5425)*, have weaker Egl and Pvl phenotypes compared with others in this category (Table 8). In both cases, vulval cells invaginate and form finger-like structures, but the overall morphology is abnormal (Figure 6, E and F). The penetrance of the vulval defect is greater in *sy5425* compared with *bh25*, although an opposite trend was observed for the Egl phenotype. We also noted that the utse is somewhat thicker in *bh25* animals (Figure 6E), although its contribution to the Egl phenotype is unclear. Interestingly, some *sy5425* animals showed ectopic P4.p and P8.p induction. This phenotype is present in the outcrossed strain, so it is either caused by the same mutation or another very closely linked mutation. More work is needed to distinguish between these two possibilities. We also observed significant embryonic and early larval lethality in the *sy5425* strain (18%, n = 284).

### *Cbr-lin-11* mutations disrupt vulva and utse morphogenesis

**Figure 7:**
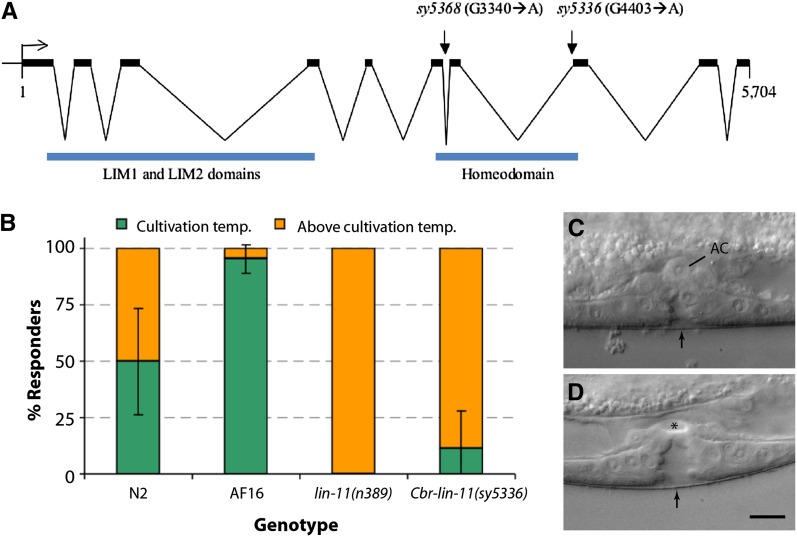
Genomic organization of *Cbr-lin-11* and mutant phenotypes. (A) *Cbr-lin-11* open reading frame showing LIM and homeodomain regions as well as mutations. Both alleles affect the homeodomain region. (B) Graph shows the thermotaxis response of the wild-type and *lin-11* animals. Wild-type animals prefer to live near the temperature at which they were initially grown, whereas *lin-11* animals do not demonstrate such behavior. Instead, they are thermophilic. (C) *lin-11(n389)*. The AC is located at the vulval apex. (D) *Cbr-lin-11(sy5336)*. No AC can be observed at the corresponding location (asterisk). Scale bar in C and D is 10 µm.

The vulva and utse defects of *lin(sy5336)* and *lin(sy5368)* animals strongly resemble those of *C. elegans lin-11* mutants (Ferguson *et al.* 1987; Newman *et al.* 1999). In addition, the thermotaxis defect of the animals is also similar [(Hobert *et al.* 1998) Figure 7B], which is reflected in their lack of preference to the cultivation temperature. Therefore, we carried out transgene rescue experiments to examine whether these two lines carry mutant alleles of *Cbr-lin-11*. Stable lines carrying a 19-kb genomic clone of *C. elegans lin-11* (*bhEx78* and *bhEx148*, on the *sy5336* genetic background) showed rescue of the vulva, utse, and Egl defects (*bhEx78*: 47% wild-type vulva and utse, n = 32, non-Egl 8%, n = 25; *bhEx148*: 71% wild-type vulva and utse, n = 31, non-Egl 12.5%, n = 48; compared with *Cbr-lin-11* mutants in Table 8). We also sequenced the *Cbr-lin-11* locus in both alleles and identified molecular changes that are predicted to disrupt splicing in the homeobox region (Figure 7A, also see *Materials and Methods*).

The aforementioned rescue experiments demonstrate that *C. elegans lin-11* can substitute for *Cbr-lin-11* function in *C. briggsae* and suggest that *lin-11* function is evolutionarily conserved. This is also supported by the analysis of the cDNA and protein sequences. The *lin-11* cDNA (*C. elegans*: 1218 bp, 10 exons; *C. briggsae*: 1239 bp, 10 exons) (see Figure S3 for *C. briggsae* sequence) is 80% conserved, and the corresponding proteins are 87% identical (94% similar).

To examine the vulval defect in *Cbr-lin-11* animals, we used the two GFP-based markers *Cbr-egl-17 (mfIs5)* and *Cbr-zmp-1 (mfIs8)*. In *C. elegans*, *egl-17* and *zmp-1* have been used extensively in cell fate specification studies (Cui and Han 2003; Gupta *et al.* 2003; Inoue *et al.* 2002). The dissection of the regulatory regions of these genes has revealed evolutionarily conserved sequences (Kirouac and Sternberg 2003). In the wild-type *C. briggsae*, the earliest expression of *Cbr-egl-17*::*gfp* is observed in mid/late-L4 stage animals in the presumptive vulC and vulD (Seetharaman *et al.* 2010). In the case of *Cbr-zmp-1*::*gfp*, GFP fluorescence is primarily observed in the presumptive vulE (Seetharaman *et al.* 2010). We found that the expression of both markers was absent in *Cbr-lin-11(sy5336)* animals (Table 9). This result supports our previous findings and a crucial role of *lin-11* in vulval cell differentiation (Gupta *et al.* 2003).

**Table 9:** Expression of vulval cell fate markers in *Cbr-lin-11* mutants

Genotype	GFP Fluorescence in Vulval Progeny	n
*mfIs5 (egl-17*::*gfp)*	vulC: 79%, vulC and vulD: 21%	113
*sy5336*; *mfIs5*	None	104
*mfIs8 (zmp-1*::*gfp)*	vulA: 2%, vulE: 91%, vulA and vulE: 7%	128
*sy5336*; *mfIs8*	None	61

GFP, green fluorescent protein; n, number of animals scored.

Despite the high conservation in *lin-11* sequence and function, we did observe an interesting difference in the AC placement between the two species. Unlike *C. elegans lin-11* animals, in which ACs fail to migrate and are located on the vulval apex in most animals (*n389*: 81.1%, n = 53; Figure 7C), no such phenotype was observed in *C. briggsae* (*sy5336*: 0%, n = 47 and *sy5368*: 0%, n = 52; Figure 7D).

## Discussion

**Figure 8:**
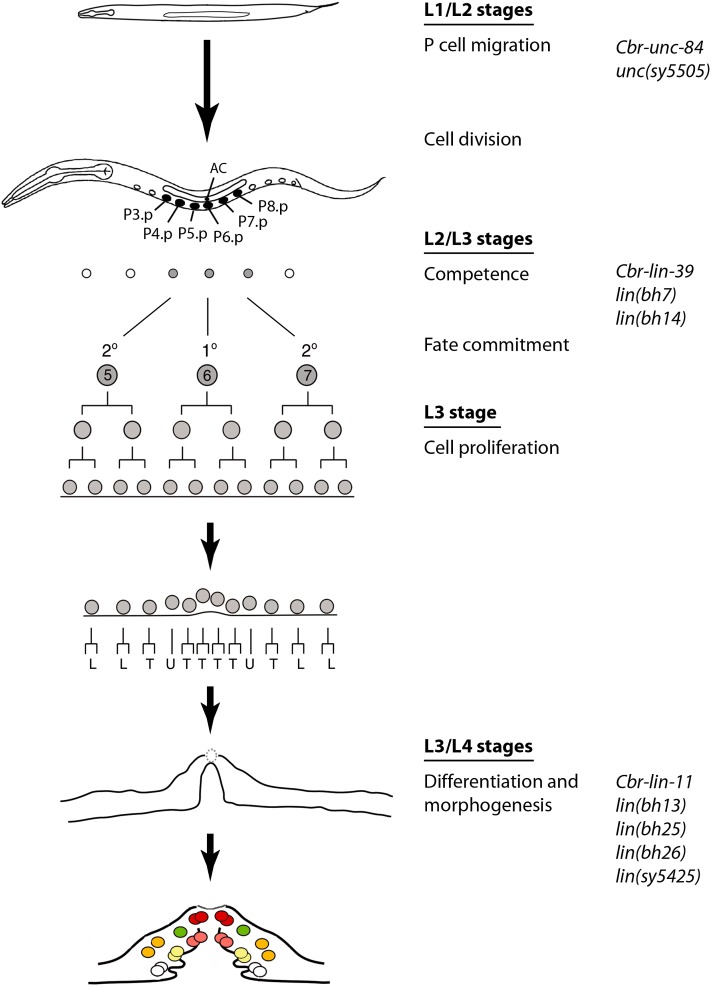
Vulval development in *C. briggsae* and the proposed roles of genes described in this study. P-cell migration into the ventral hypodermal region is mediated by the class 2 genes. Subsequently, P cells divide, and six of their posterior daughters (Pn.p, n = 3−8), termed VPCs, become capable of giving rise to the vulval tissue. Their competence appears to be regulated by the class 3 genes. VPC progeny differentiate and undergo morphogenetic changes during the L3 and L4 stages. The class 4 genes are required in these processes.

We report the isolation and characterization of mutations in 13 genes in *C. briggsae* that are involved in the development and function of the egg-laying system. To date, this is the largest set of genes identified by a forward genetics approach in this species. Ten of these genes are involved in various steps of vulval development (Figure 8). Transgene rescue and molecular analyses have revealed that three genes are orthologs of *unc-84*, *lin-39*, and *lin-11*. Together, these mutant strains serve as valuable tools for comparative and evolutionary studies. Another genetic screen in *C. briggsae* was previously carried out to identify dauer pathway genes (Inoue *et al.* 2007). The screen identified several mutations, including alleles of *Cbr-daf-2* (insulin receptor), *Cbr-daf-3* (Smad), and *Cbr-daf-4* (TGF-β family receptor). Genetic studies revealed that although the functions of *C. elegans* orthologs are conserved in *C. briggsae*, the two species exhibit differences in their temperature sensitivities. Thus, comparative genetics approaches are useful for revealing the similarities and differences in biological processes between *C. elegans* and *C. briggsae*.

Egl phenotype-based genetic screens were first carried out in *C. elegans* and led to the identification of many genes involved in vulval development (Ferguson and Horvitz 1985; Trent *et al.* 1983). Characterization of their function revealed a genetic pathway for the formation of the vulva (Ferguson *et al.* 1987). During the past decade and a half, this knowledge has been extended to other distant species, such as *P. pacificus* and *O. tipulae*, resulting in a better understanding of the evolutionary changes in the mechanism of vulva formation. Screens in *O. tipulae* have identified several mutations affecting vulva formation. Although the majority of these cause defects in VPC division and competence (Dichtel *et al.* 2001; Louvet-Vallee *et al.* 2003), others affecting vulva centering and hyper- and hypo-induced phenotypes also have been identified (Dichtel *et al.* 2001). Similar mutant classes have been found in *P. pacificus* screens as well (Eizinger *et al.* 1999; Sommer 2005). Compared with *C. elegans*, the phenotypic spectrum in these two species is quite different. For example, mutations affecting Pn.p fate (*e.g.*, 1° converted to 2° or 3°) were recovered quite frequently in *C. elegans* but not in *O. tipulae* or *P. pacificus*. Interestingly, our screens in *C. briggsae* also revealed differences from *C. elegans*. We did not find Pn.p fate mutants, and one-half of the mutants examined show defects in cell invagination and morphogenesis (Table 8, Figure 8). It is not clear whether this phenotypic distribution is typical in *C. briggsae* or whether it results from the lack of saturation in the screen or another reason. It is worth pointing out that in our experience, Egl animals in *C. briggsae* are more difficult to identify in a standard F2 screen in the presence of predominantly non-Egl worms. This may be at least partly caused by the tendency of *C. briggsae* to retain fewer eggs in the uterus compared to *C. elegans* (B. P. Gupta, unpublished results; T. Inoue and M. A. Felix, personal communications). The difficulty in isolating Egls could have limited the recovery of vulva-specific mutants and the phenotypic classes in our screens. Future genetic screens using different approaches will help to address this issue. Furthermore, combining a vulva-specific GFP reporter with an Egl phenotype may be a useful approach, as it will minimize the recovery of non-vulval mutations.

### Phenotypic classes recovered in our screen

We have isolated four phenotypic classes of worms, all of which affect egg laying. Mutations in one of these (class 1) have normal vulva and utse morphologies. To investigate the role of sex muscles and HSNs, we used serotonin and fluoxetine drugs to induce egg laying. *sy5395* mutants showed a mild but obvious increase in egg laying in response to serotonin, suggesting that the muscle function is intact. However, *bh2* and *bh21* did not respond to any of the drugs, suggesting that muscle function may be impaired.

The class 2 mutations *sy5505* and *sy5506* affect nuclear migration in a temperature-dependent manner. Both alleles cause animals to develop Egl and Unc phenotypes due to the failure of P nuclei to migrate into the ventral cord region. The phenotype of *unc(sy5506)* animals can be rescued by a *C. elegans unc-84* transgene, suggesting that *sy5506* is an allele of *Cbr-unc-84*. This conclusion is also supported by our mapping and allele sequencing data. The remaining two classes of mutations alter the number of vulval progeny and vulval invagination. *Cbr-lin-39* is required for the maintenance of VPC competence and appears to act downstream of *Cbr-pry-1/axin*-mediated Wnt signaling. *Cbr-lin-11* controls vulval cell differentiation and tissue morphogenesis. Both genes belong to conserved families of transcription factors (the *Dfd/Scr*-related Hox family and the *LIM-Hox* family, respectively).

### Nuclear migration in *C. briggsae* is mediated by a conserved SUN domain protein

Nuclear migration plays important roles in diverse cellular processes, including cell division, cell polarity and cell migration. In *C. elegans*, well-studied nuclear migration events are observed with the hyp7 syncytium (dorsal side of the hypodermis) and a set of hypodermal blast cells (P cells) that form the vulva. Genetic analysis of these events has revealed that UNC-83 (KASH domain) and UNC-84 (SUN domain) proteins form a bridge-like structure to connect the nucleus to microtubules, motor proteins, and other cytoskeletal components. The movement of Kinesin and Dynein motors in a coordinated manner causes the nucleus to move in a specific direction. The vulval defects in *C. briggsae sy5505* and *sy5506* animals are typical of *unc-83* and *unc-84* mutants. Both alleles exhibit a temperature-sensitive phenotype and display Egl and Unc phenotypes. The phenotypes of *sy5506* animals can be efficiently rescued by a *C. elegans unc-84* genomic clone, which demonstrates that *unc-84* plays a conserved role in nuclear migration in both species.

### Genes affecting cell fusion in *C. briggsae*

We isolated two alleles of *Cbr-lin-39*, both of which prevent VPC induction. In *C. elegans*, P(3-8).p escape fusion in the L1 and L2 stages and remain competent to respond to induction during the L3 stage (Sternberg 2005). This process is regulated by *lin-39* (Clark *et al.* 1993). The expression of *lin-39* at the L2 stage appears to be partly controlled by BAR-1/β-catenin−mediated Wnt signaling because in *bar-1* mutants, *lin-39* expression in VPCs is reduced, resulting in fusion of some VPCs to the hyp7 syncytium (Eisenmann *et al.* 1998). Our experiments on *Cbr-lin-39* suggest that the function of *lin-39* in VPC competence is conserved in *C. briggsae*. This finding is supported by the analysis of mutant phenotype, rescue experiments, cell fusion studies using the *dlg-1*::*GFP* marker, and genetic interaction with *Cbr-pry-1* (Axin family). Our previous results involving RNA interference−mediated knockdown of *Cbr-lin-39* (Seetharaman *et al.* 2010) also support these findings.

*lin-39* orthologs also have been identified in *O. tipulae* and *P. pacificus*. *Oti-lin-39* appears to control VPC competence by preventing fusion of Pn.p cells in the late-L1/early-L2 stages (Louvet-Vallee *et al.* 2003). However, the function of *Ppa-lin-39* appears to have diverged. In *Ppa-lin-39* mutants, VPCs undergo programmed cell death instead of fusing with the hypodermis (Eizinger and Sommer 1997). Taken together, these findings suggest that although *lin-39* function is conserved in *Caenorhabditis* and *Oscheius* species, it has acquired new roles in *Pristionchus*. In the future, analysis of the role of *lin-39* in additional nematode species will allow for a more detailed comparison of its roles in vulval development.

In addition to *Cbr-lin-39*, we have uncovered two other loci, *lin(bh7)* and *lin(bh14)*, that control VPC competence. The phenotype of both mutants is weaker than that of *bh20* animals, perhaps due to weak hypomorphic alleles. Alternatively, these genes may have some redundant function. More alleles are required to distinguish between these two possibilities. The induction of P5.p and P7.p in *bh7* and *bh14* animals is frequently affected. To test the induction potential of these two VPCs, we carried out two complementary experiments. We examined their pattern of division after ablation of P6.p. Furthermore, the effect of *lin-3* overexpression was investigated. We found that in the absence of P6.p, the P5.p and P7.p cells were induced to various extents, suggesting that these VPCs can respond to inductive signal. This conclusion is strongly supported by the *lin-3* dosage experiments. High doses of *lin-3* during the L2 stage caused ectopic VPC induction, resulting in a Muv phenotype. We can therefore conclude that class 3 genes interact with a LIN-3-like inductive signal to regulate VPC competence in *C. briggsae*.

### *lin-11* is a key regulator of vulval morphogenesis

*lin-11* is a founding member of the LIM homeobox family of genes. Mutations in *lin-11* were originally isolated in genetic screens for worms that failed to lay eggs (Ferguson and Horvitz 1985). Subsequently, phenotypic analyses showed a wide-range of defects affecting vulval morphology (Freyd *et al.* 1990; Gupta *et al.* 2003), utse formation (Newman *et al.* 1999), and neuronal differentiation (Hobert *et al.* 1998; Sarafi-Reinach *et al.* 2001). *C. briggsae lin-11* mutants exhibit defects in the egg-laying system similar to those observed in *C. elegans lin-11* animals. Thus, vulval cells fail to invaginate, and a functional connection between the vulva and the uterus is not established. These phenotypes can be rescued by a *C. elegans lin-11* genomic fragment, suggesting that *lin-11* regulatory and coding sequences are evolutionarily conserved. This supports our previous conclusions on the conservation of *lin-11* regulation by Wnt and LIN-12/Notch signaling pathways in the vulva and π cell differentiation (Marri and Gupta 2009).

Outside the reproductive system, *lin-11* also is involved in the differentiation of several olfactory and chemosensory neurons (Sarafi-Reinach *et al.* 2001). We have not yet characterized the neuronal role of *Cbr-lin-11* in detail, but we have found that *Cbr-lin-11* mutants have a thermotaxis defect similar to that reported in *C. elegans lin-11* animals. Thus, similar to the egg-laying system, the role of *lin-11* in thermosensory behavior is also conserved. The recovery of *Cbr-lin-11* alleles provides a unique opportunity to investigate the mechanism of cell differentiation in *C. briggsae* and *C. elegans*.

### DSD in *C. briggsae* vulva formation

Kiontke *et al.* 2007 had earlier reported variations in several steps of vulva formation in Rhabditid species. These included changes in Pn.p cell competence, cell division pattern, and vulva position. The phenotypic analysis of *C. briggsae* vulva mutants and molecular cloning of the 3 loci in which the mutations are located has revealed DSD in homologous processes. We observed interesting differences in at least three cases. First, the P5.p-P6.p inter-VPC distance in *lin-39* mutants tends to be lower in *C. briggsae* than in *C. elegans*. Second, *lin-39* does not interact with *pry-1* to enhance the small nuclear size phenotype of posterior Pn.p cells in *C. briggsae* as it does in *C. elegans* (Penigault and Felix 2011b). Finally, *Cbr-lin-11* animals do not show AC migration defects, which is one of the hallmarks of *lin-11* mutants in *C. elegans*. These results reveal the differences in developmental mechanisms that exist despite conservation of vulval morphology in these two species.

### *C. briggsae* as a model for the study of vulval development

*C. briggsae* is increasingly being used in comparative developmental and evolutionary studies. In recent years, a number of publications have described processes such as sex determination (Guo *et al.* 2009; Hill *et al.* 2006; Kelleher *et al.* 2008), dauer formation (Inoue *et al.* 2007), pheromone receptor signaling (McGrath *et al.* 2011), embryogenesis (Lin *et al.* 2009; Zhao *et al.* 2008), and vulva formation in *C. briggsae* (Felix 2007; Hoyos *et al.* 2011; Marri and Gupta 2009; Penigault and Felix 2011a; Seetharaman *et al.* 2010). The findings have revealed similarities and differences in developmental processes.

The analysis of the vulval precursor fates in *C. briggsae* has revealed the role of conserved signaling pathway genes such as Ras, Notch, and Wnt. However, a detailed examination of gene function and pathways could not be carried out due to the lack of mutations affecting specific steps in the vulval development process. The mutations described in this study represent the first systematic effort in *C. briggsae* to investigate the genetic basis of vulva formation. Future work is needed to reveal the mechanism of gene function and to further compare *C. briggsae* to *C. elegans*. The results will ultimately help clarify how distinct processes form almost identical vulval structures in these two species.

## Supplementary Material

Supporting Information

## References

[bib1] Antoshechkin I. Sternberg P. W., 2007 The versatile worm: genetic and genomic resources for *Caenorhabditis elegans* research. Nat. Rev. Genet. 8: 518–532 17549065 10.1038/nrg2105

[bib2] Avery L. Horvitz H. R., 1987 A cell that dies during wild-type *C. elegans* development can function as a neuron in a *ced-3* mutant. Cell 51: 1071–1078 3690660 10.1016/0092-8674(87)90593-9 PMC3773210

[bib3] Baldessarini R. J., 1996 Drugs and the treatment of psychiatric disorders: antimanic and antidepressant agents, pp. 249–263 in Goodman and Gilman’s Pharmacological Basis of Therapeutics, Ed. 12, edited by Brunton L. L. Chabner B. A. Knollmann B. C. McGraw-Hill, New York

[bib4] Benian G. M. L’Hernault S. W. Morris M. E., 1993 Additional sequence complexity in the muscle gene, unc-22, and its encoded protein, twitchin, of *Caenorhabditis elegans*. Genetics 134: 1097–1104 8397135 10.1093/genetics/134.4.1097 PMC1205578

[bib81] Brenner S., 1974 The genetics of *Caenorhabditis elegans*. Genetics 77: 71–94 4366476 10.1093/genetics/77.1.71 PMC1213120

[bib5] Burdine R. D. Stern M. J., 1996 Easy RNA isolation from *C. elegans*: a TRIZOL based method. Worm Breed. Gaz. 14: 10

[bib6] Clark S. G. Chisholm A. D. Horvitz H. R., 1993 Control of cell fates in the central body region of *C. elegans* by the homeobox gene lin-39. Cell 74: 43–55 8101475 10.1016/0092-8674(93)90293-y

[bib7] Croll N. A., 1975 Indolealkylamines in the coordination of nematode behavioral activities. Can. J. Zool. 53: 894–903 1079747 10.1139/z75-103

[bib8] Cui M. Han M., 2003 Cis regulatory requirements for vulval cell-specific expression of the *Caenorhabditis elegans* fibroblast growth factor gene egl-17. Dev. Biol. 257: 104–116 12710960 10.1016/s0012-1606(03)00033-2

[bib9] Cutter A. D., 2008 Divergence times in *Caenorhabditis* and *Drosophila* inferred from direct estimates of the neutral mutation rate. Mol. Biol. Evol. 25: 778–786 18234705 10.1093/molbev/msn024

[bib10] Delattre M. Felix M. A., 2001 Polymorphism and evolution of vulval precursor cell lineages within two nematode genera, *Caenorhabditis* and *Oscheius*. Curr. Biol. 11: 631–643 11369226 10.1016/s0960-9822(01)00202-0

[bib11] Dempsey C. M. Mackenzie S. M. Gargus A. Blanco G. Sze J. Y., 2005 Serotonin (5HT), fluoxetine, imipramine and dopamine target distinct 5HT receptor signaling to modulate *Caenorhabditis elegans* egg-laying behavior. Genetics 169: 1425–1436 15654117 10.1534/genetics.104.032540 PMC1449529

[bib12] Dichtel M. L. Louvet-Vallee S. Viney M. E. Felix M. A. Sternberg P. W., 2001 Control of vulval cell division number in the nematode Oscheius/Dolichorhabditis sp. CEW1. Genetics 157: 183–197 11139501 10.1093/genetics/157.1.183 PMC1461485

[bib13] Eisenmann D. M., 2005 Wnt signaling (June 25, 2005). WormBook, ed. The *C. elegans* Research Community WormBook, doi/10.1895/wormbook.1.7.1, http://www.wormbook.org 10.1895/wormbook.1.7.1 PMC4781570 18050402

[bib14] Eisenmann D. M. Maloof J. N. Simske J. S. Kenyon C. Kim S. K., 1998 The beta-catenin homolog BAR-1 and LET-60 Ras coordinately regulate the Hox gene *lin-39* during *Caenorhabditis elegans* vulval development. Development 125: 3667–3680 9716532 10.1242/dev.125.18.3667

[bib15] Eizinger A. Sommer R. J., 1997 The homeotic gene *lin-39* and the evolution of nematode epidermal cell fates. Science 278: 452–455 9334302 10.1126/science.278.5337.452

[bib16] Eizinger A. Jungblut B. Sommer R. J., 1999 Evolutionary change in the functional specificity of genes. Trends Genet. 15: 197–202 10322487 10.1016/s0168-9525(99)01728-x

[bib17] Felix M. A., 2007 Cryptic quantitative evolution of the vulva intercellular signaling network in *Caenorhabditis*. Curr. Biol. 17: 103–114 17240335 10.1016/j.cub.2006.12.024

[bib18] Felix M. A. Sternberg P. W., 1997 Two nested gonadal inductions of the vulva in nematodes. Development 124: 253–259 9006085 10.1242/dev.124.1.253

[bib19] Felix M. A. De Ley P. Sommer R. J. Frisse L. Nadler S. A., 2000 Evolution of vulva development in the Cephalobina (Nematoda). Dev. Biol. 221: 68–86 10772792 10.1006/dbio.2000.9665

[bib20] Ferguson E. L. Horvitz H. R., 1985 Identification and characterization of 22 genes that affect the vulval cell lineages of the nematode *Caenorhabditis elegans*. Genetics 110: 17–72 3996896 10.1093/genetics/110.1.17 PMC1202554

[bib21] Ferguson E. L. Sternberg P. W. Horvitz H. R., 1987 A genetic pathway for the specification of the vulval cell lineages of *Caenorhabditis elegans*. Nature 326: 259–267 2881214 10.1038/326259a0

[bib22] Flibotte S. Edgley M. L. Maydan J. Taylor J. Zapf R., 2009 Rapid high resolution single nucleotide polymorphism-comparative genome hybridization mapping in *Caenorhabditis elegans*. Genetics 181: 33–37 18957702 10.1534/genetics.108.096487 PMC2621182

[bib23] Freyd G., 1991 Molecular analysis of the *Caenorhabditis elegans* cell lineage gene lin-11. Ph.D. Thesis, Massachusetts Institute of Technology, Boston

[bib24] Freyd G. Kim S. K. Horvitz H. R., 1990 Novel cysteine-rich motif and homeodomain in the product of the *Caenorhabditis elegans* cell lineage gene lin-11. Nature 344: 876–879 1970421 10.1038/344876a0

[bib25] Gleason J. E. Korswagen H. C. Eisenmann D. M., 2002 Activation of Wnt signaling bypasses the requirement for RTK/Ras signaling during *C. elegans* vulval induction. Genes Dev. 16: 1281–1290 12023306 10.1101/gad.981602 PMC186276

[bib26] Gleason J. E. Szyleyko E. A. Eisenmann D. M., 2006 Multiple redundant Wnt signaling components function in two processes during *C. elegans* vulval development. Dev. Biol. 298: 442–457 16930586 10.1016/j.ydbio.2006.06.050

[bib27] Greenwald I., 2005 LIN-12/Notch signaling in *C. elegans* (August 4, 2005). WormBook, ed. The *C. elegans* Research Community WormBook, doi/10.1895/wormbook.1.10.1, http://www.wormbook.org

[bib28] Guo Y. Lang S. Ellis R. E., 2009 Independent recruitment of F box genes to regulate hermaphrodite development during nematode evolution. Curr. Biol. 19: 1853–1860 19836240 10.1016/j.cub.2009.09.042

[bib29] Gupta B. P. Sternberg P. W., 2003 The draft genome sequence of the nematode *Caenorhabditis briggsae*, a companion to *C. elegans*. Genome Biol. 4: 238 14659008 10.1186/gb-2003-4-12-238 PMC329410

[bib30] Gupta B. P. Wang M. Sternberg P. W., 2003 The C. elegans LIM homeobox gene lin-11 specifies multiple cell fates during vulval development. Development 130: 2589–2601 12736204 10.1242/dev.00500

[bib31] Gupta B. P. Liu J. Hwang B. J. Moghal N. Sternberg P. W., 2006 sli-3 negatively regulates the LET-23/epidermal growth factor receptor-mediated vulval induction pathway in *Caenorhabditis elegans*. Genetics 174: 1315–1326 16980384 10.1534/genetics.106.063990 PMC1667086

[bib32] Gupta B. P. Johnsen R. Chen N., 2007 Genomics and biology of the nematode *Caenorhabditis briggsae* (May 3, 2007). WormBook, ed. The *C. elegans* Research Community WormBook, doi/10.1895/wormbook.1.10.1, http://www.wormbook.org 10.1895/wormbook.1.136.1 PMC4781269 18050493

[bib33] Hill R. J. Sternberg P. W., 1992 The gene lin-3 encodes an inductive signal for vulval development in *C. elegans*. Nature 358: 470–476 1641037 10.1038/358470a0

[bib34] Hill R. C. de Carvalho C. E. Salogiannis J. Schlager B. Pilgrim D., 2006 Genetic flexibility in the convergent evolution of hermaphroditism in *Caenorhabditis nematodes*. Dev. Cell 10: 531–538 16580997 10.1016/j.devcel.2006.02.002

[bib35] Hillier L. W. Miller R. D. Baird S. E. Chinwalla A. Fulton L. A., 2007 Comparison of *C. elegans* and *C. briggsae* genome sequences reveals extensive conservation of chromosome organization and synteny. PLoS Biol. 5: e167 17608563 10.1371/journal.pbio.0050167 PMC1914384

[bib36] Hobert O. D’Alberti T. Liu Y. Ruvkun G., 1998 Control of neural development and function in a thermoregulatory network by the LIM homeobox gene lin-11. J. Neurosci. 18: 2084–2096 9482795 10.1523/JNEUROSCI.18-06-02084.1998 PMC6792926

[bib37] Horvitz H. R. Sulston J. E., 1980 Isolation and genetic characterization of cell-lineage mutants of the nematode *Caenorhabditis elegans*. Genetics 96: 435–454 7262539 10.1093/genetics/96.2.435 PMC1214309

[bib38] Horvitz H. R. Chalfie M. Trent C. Sulston J. E. Evans P. D., 1982 Serotonin and octopamine in the nematode *Caenorhabditis elegans*. Science 216: 1012–1014 6805073 10.1126/science.6805073

[bib39] Hoyos E. Kim K. Milloz J. Barkoulas M. Penigault J. B., 2011 Quantitative variation in autocrine signaling and pathway crosstalk in the *Caenorhabditis* vulval network. Curr. Biol. 21: 527–538 21458263 10.1016/j.cub.2011.02.040 PMC3084603

[bib40] Inoue T. Sherwood D. R. Aspock G. Butler J. A. Gupta B. P., 2002 Gene expression markers for *Caenorhabditis elegans* vulval cells. Mech. Dev. 119(Suppl 1): S203–S209 14516686 10.1016/s0925-4773(03)00117-5

[bib41] Inoue T. Ailion M. Poon S. Kim H. K. Thomas J. H., 2007 Genetic analysis of dauer formation in *Caenorhabditis briggsae*. Genetics 177: 809–818 17660533 10.1534/genetics.107.078857 PMC2034645

[bib42] Katz W. S. Hill R. J. Clandinin T. R. Sternberg P. W., 1995 Different levels of the *C. elegans* growth factor LIN-3 promote distinct vulval precursor fates. Cell 82: 297–307 7628018 10.1016/0092-8674(95)90317-8

[bib43] Kelleher D. F. de Carvalho C. E. Doty A. V. Layton M. Cheng A. T., 2008 Comparative genetics of sex determination: masculinizing mutations in *Caenorhabditis briggsae*. Genetics 178: 1415–1429 18245372 10.1534/genetics.107.073668 PMC2278099

[bib44] Kimble J., 1981 Alterations in cell lineage following laser ablation of cells in the somatic gonad of *Caenorhabditis elegans*. Dev. Biol. 87: 286–300 7286433 10.1016/0012-1606(81)90152-4

[bib45] Kiontke K. Barriere A. Kolotuev I. Podbilewicz B. Sommer R., 2007 Trends, stasis, and drift in the evolution of nematode vulva development. Curr. Biol. 17: 1925–1937 18024125 10.1016/j.cub.2007.10.061

[bib46] Kirouac M. Sternberg P. W., 2003 cis-Regulatory control of three cell fate-specific genes in vulval organogenesis of *Caenorhabditis elegans* and *C. briggsae*. Dev. Biol. 257: 85–103 12710959 10.1016/s0012-1606(03)00032-0

[bib47] Koboldt D. C. Staisch J. Thillainathan B. Haines K. Baird S. E., 2010 A toolkit for rapid gene mapping in the nematode *Caenorhabditis briggsae*. BMC Genomics 11: 236 20385026 10.1186/1471-2164-11-236 PMC2864247

[bib48] Li C. Chalfie M., 1990 Organogenesis in *C. elegans*: positioning of neurons and muscles in the egg-laying system. Neuron 4: 681–695 2344407 10.1016/0896-6273(90)90195-l

[bib49] Lin K. T. Broitman-Maduro G. Hung W. W. Cervantes S. Maduro M. F., 2009 Knockdown of SKN-1 and the Wnt effector TCF/POP-1 reveals differences in endomesoderm specification in *C. briggsae* as compared with *C. elegans*. Dev. Biol. 325: 296–306 18977344 10.1016/j.ydbio.2008.10.001 PMC2648516

[bib50] Louvet-Vallee S. Kolotuev I. Podbilewicz B. Felix M. A., 2003 Control of vulval competence and centering in the nematode *Oscheius* sp. 1 CEW1. Genetics 163: 133–146 12586702 10.1093/genetics/163.1.133 PMC1462419

[bib51] Malone C. J. Fixsen W. D. Horvitz H. R. Han M., 1999 UNC-84 localizes to the nuclear envelope and is required for nuclear migration and anchoring during *C. elegans* development. Development 126: 3171–3181 10375507 10.1242/dev.126.14.3171

[bib52] Maloof J. N. Kenyon C., 1998 The Hox gene lin-39 is required during *C. elegans* vulval induction to select the outcome of Ras signaling. Development 125: 181–190 9486792 10.1242/dev.125.2.181

[bib53] Marri S. Gupta B. P., 2009 Dissection of lin-11 enhancer regions in *Caenorhabditis elegans* and other nematodes. Dev. Biol. 325: 402–411 18950616 10.1016/j.ydbio.2008.09.026

[bib54] Maydan J. S. Flibotte S. Edgley M. L. Lau J. Selzer R. R., 2007 Efficient high-resolution deletion discovery in *Caenorhabditis elegans* by array comparative genomic hybridization. Genome Res. 17: 337–347 17267812 10.1101/gr.5690307 PMC1800925

[bib55] McGee M. D. Rillo R. Anderson A. S. Starr D. A., 2006 UNC-83 IS a KASH protein required for nuclear migration and is recruited to the outer nuclear membrane by a physical interaction with the SUN protein UNC-84. Mol. Biol. Cell 17: 1790–1801 16481402 10.1091/mbc.E05-09-0894 PMC1415293

[bib56] McGrath P. T. Xu Y. Ailion M. Garrison J. L. Butcher R. A., 2011 Parallel evolution of domesticated Caenorhabditis species targets pheromone receptor genes. Nature 477: 321–325 21849976 10.1038/nature10378 PMC3257054

[bib57] Mello C. C. Kramer J. M. Stinchcomb D. Ambros V., 1991 Efficient gene transfer in *C.elegans*: extrachromosomal maintenance and integration of transforming sequences. EMBO J. 10: 3959–3970 1935914 10.1002/j.1460-2075.1991.tb04966.x PMC453137

[bib58] Miller M. A. Cutter A. D. Yamamoto I. Ward S. Greenstein D., 2004 Clustered organization of reproductive genes in the *C. elegans* genome. Curr. Biol. 14: 1284–1290 15268860 10.1016/j.cub.2004.07.025

[bib59] Myers T. R. Greenwald I., 2007 Wnt signal from multiple tissues and lin-3/EGF signal from the gonad maintain vulval precursor cell competence in *Caenorhabditis elegans*. Proc. Natl. Acad. Sci. USA 104: 20368–20373 18077322 10.1073/pnas.0709989104 PMC2154437

[bib60] Newman A. P. Sternberg P. W., 1996 Coordinated morphogenesis of epithelia during development of the *Caenorhabditis elegans* uterine-vulval connection. Proc. Natl. Acad. Sci. USA 93: 9329–9333 8790329 10.1073/pnas.93.18.9329 PMC38427

[bib61] Newman A. P. Acton G. Z. Hartwieg E. Horvitz H. R. Sternberg P. W., 1999 The lin-11 LIM domain transcription factor is necessary for morphogenesis of *C. elegans* uterine cells. Development 126: 5319–5326 10556057 10.1242/dev.126.23.5319

[bib62] Penigault J. B. Felix M. A., 2011a Evolution of a system sensitive to stochastic noise: P3.p cell fate in Caenorhabditis. Dev. Biol. 357: 419–427 21693113 10.1016/j.ydbio.2011.05.675

[bib63] Penigault J. B. Felix M. A., 2011b High sensitivity of *C. elegans* vulval precursor cells to the dose of posterior Wnts. Dev. Biol. 357: 428–438 21708144 10.1016/j.ydbio.2011.06.006

[bib64] Rudel D. Kimble J., 2001 Conservation of glp-1 regulation and function in nematodes. Genetics 157: 639–654 11156985 10.1093/genetics/157.2.639 PMC1461503

[bib65] Sarafi-Reinach T. R. Melkman T. Hobert O. Sengupta P., 2001 The lin-11 LIM homeobox gene specifies olfactory and chemosensory neuron fates in C. elegans. Development 128: 3269–3281 11546744 10.1242/dev.128.17.3269

[bib66] Seetharaman A. Cumbo P. Bojanala N. Gupta B. P., 2010 Conserved mechanism of Wnt signaling function in the specification of vulval precursor fates in *C. elegans* and *C. briggsae*. Dev. Biol. 346: 128–139 20624381 10.1016/j.ydbio.2010.07.003

[bib67] Sommer R. J., 2005 Evolution of development in nematodes related to *C. elegans* (December 14, 2005). WormBook, ed. The *C. elegans* Research Community WormBook, doi/10.1895/wormbook.1.10.1, http://www.wormbook.org 10.1895/wormbook.1.46.1 PMC4781599 18050392

[bib68] Sommer R. J. Sternberg P. W., 1996 Evolution of nematode vulval fate patterning. Dev. Biol. 173: 396–407 8606000 10.1006/dbio.1996.0035

[bib69] Sommer R. J. Carta L. K. Sternberg P. W., 1994 The evolution of cell lineage in nematodes. Dev. Suppl. 1994: 85–95 7579527

[bib70] Starr D. A., 2011 Watching nuclei move: insights into how kinesin-1 and dynein function together. BioArchitecture 1: 9–13 21866255 10.4161/bioa.1.1.14629 PMC3158635

[bib71] Starr D. A. Hermann G. J. Malone C. J. Fixsen W. Priess J. R., 2001 *unc-83* encodes a novel component of the nuclear envelope and is essential for proper nuclear migration. Development 128: 5039–5050 11748140 10.1242/dev.128.24.5039

[bib72] Stein L. D. Bao Z. Blasiar D. Blumenthal T. Brent M. R., 2003 The genome sequence of *Caenorhabditis briggsae*: a platform for comparative genomics. PLoS Biol. 1: E45 14624247 10.1371/journal.pbio.0000045 PMC261899

[bib73] Sternberg P. W., 2005 Vulval development (June 25, 2005). WormBook, ed. The *C. elegans* Research Community WormBook, doi/10.1895/wormbook.1.10.1, http://www.wormbook.org 10.1895/wormbook.1.6.1 PMC4781130 18050418

[bib74] Sternberg P. W. Horvitz H. R., 1986 Pattern formation during vulval development in *C. elegans*. Cell 44: 761–772 3753901 10.1016/0092-8674(86)90842-1

[bib82] Sulston J. E., 1976 Post-embryonic development in the ventral cord of Caenorhabditis elegans. Philos. Trans. R. Soc. Lond. B. Biol. Sci. 275: 287–297 8804 10.1098/rstb.1976.0084

[bib75] Sulston J. E. Horvitz H. R., 1981 Abnormal cell lineages in mutants of the nematode *Caenorhabditis elegans*. Dev. Biol. 82: 41–55 7014288 10.1016/0012-1606(81)90427-9

[bib83] Sulston J. E. White J. G., 1980 Regulation and cell autonomy during postembryonic development of Caenorhabditis elegans. Dev. Biol. 78: 577–597 7190941 10.1016/0012-1606(80)90353-x

[bib76] Trent C. Tsuing N. Horvitz H. R., 1983 Egg-laying defective mutants of the nematode *Caenorhabditis elegans*. Genetics 104: 619–647 11813735 10.1093/genetics/104.4.619 PMC1202130

[bib77] True J. Haag E., 2001 Developmental system drift and flexibility in evolutionary trajectories. Evol. Dev. 3: 109–119 11341673 10.1046/j.1525-142x.2001.003002109.x

[bib78] Weinshenker D. Garriga G. Thomas J. H., 1995 Genetic and pharmacological analysis of neurotransmitters controlling egg laying in *C. elegans*. J. Neurosci. 15: 6975–6985 7472454 10.1523/JNEUROSCI.15-10-06975.1995 PMC6577982

[bib84] Wood W. B., 1988 The nematode Caenorhabditis elegans. Cold Spring Harbor Laboratory Press, New York

[bib79] Zhao Z. Boyle T. J. Bao Z. Murray J. I. Mericle B., 2008 Comparative analysis of embryonic cell lineage between *Caenorhabditis briggsae* and *Caenorhabditis elegans*. Dev. Biol. 314: 93–99 18164284 10.1016/j.ydbio.2007.11.015 PMC2696483

[bib80] Zhao Z. Flibotte S. Murray J. I. Blick D. Boyle T. J., 2010 New tools for investigating the comparative biology of *Caenorhabditis briggsae* and *C. elegans*. Genetics 184: 853–863 20008572 10.1534/genetics.109.110270 PMC2845351

