# Supplementary material for: Genetic Control of Vulval Development in Caenorhabditis briggsae
Source: G3 (Bethesda). 2012 Dec 1;2(12):1625–41. doi: 10.1534/g3.112.004598 (PMC3516484; doi:10.1534/g3.112.004598)
Supplement: Supporting Information [file supp_2_12_1625__index.html]

Supporting Information 

# Genetic Control of Vulval Development in *Caenorhabditis briggsae*

## Supporting Information for Sharanya *et al.*, 2012

**Files in this Data Supplement:**

- Supporting Information - Figures S1-S3 and Table S1 (PDF, 6 MB)
- Figure S1 - Mutation mapping using indel and snip-SNP polymorphisms (PDF, 782 KB)
- Figure S2 - Mutation mapping using SNP-chip (PDF, 4 MB)
- Figure S3 - *Cbr-lin-11* cDNA sequence (PDF, 1.3 MB)
- Table S1 - List of PCR and sequencing primers used in this study (PDF, 59 KB)
