# Supplementary material for: Genetic Control of Vulval Development in Caenorhabditis briggsae
Source: G3 (Bethesda). 2012 Dec 1;2(12):1625–41. doi: 10.1534/g3.112.004598 (PMC3516484; doi:10.1534/g3.112.004598)
Supplement: Supporting Information [file supp_2.12.1625_004598SI.pdf]

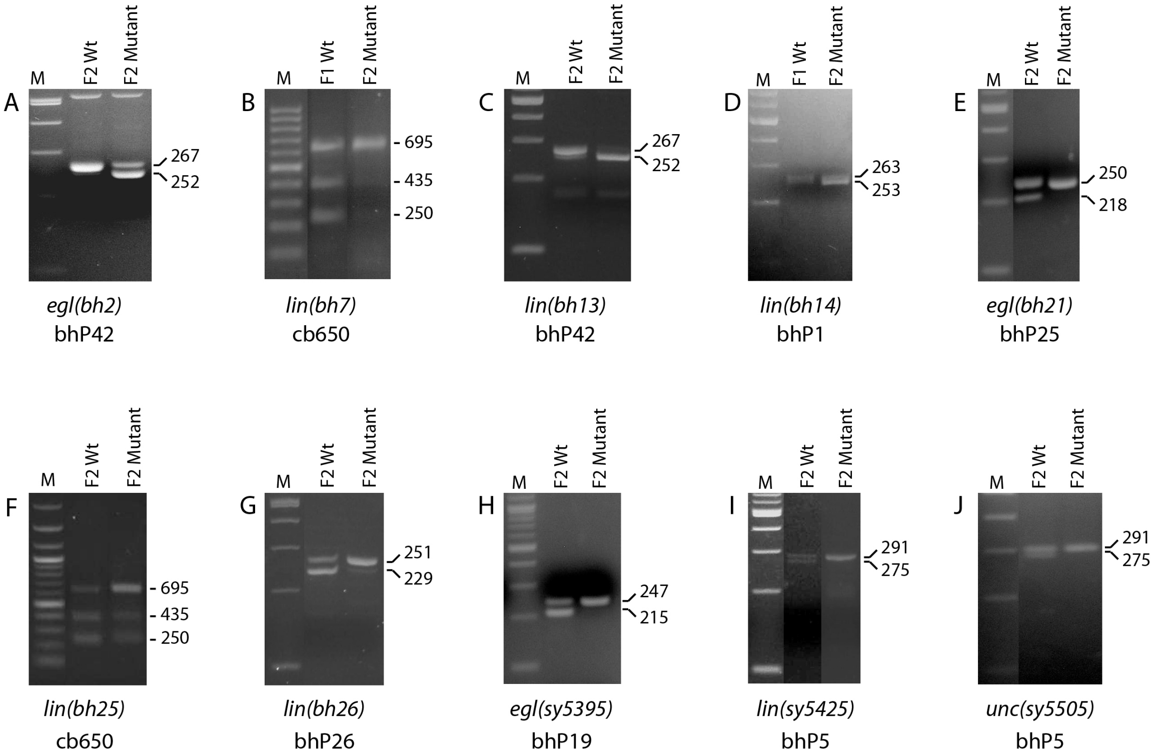

Figure S1 Mutation mapping using indel and snip-SNP polymorphisms. The images show DNA bands on 4% (for indels) and 1% (for snip-SNPs) agarose gels. The polymorphisms have been previously described (Koboldt *et al.* 2010). See Materials and Methods and Table 3 for details. M: DNA molecular weight marker, F1 Wt: DNA from F1 heterozygous animals, F2 Wt: DNA from phenotypically wild type pool of F2 animals, F2 Mutant: DNA from phenotypically mutant pool of F2 animals.

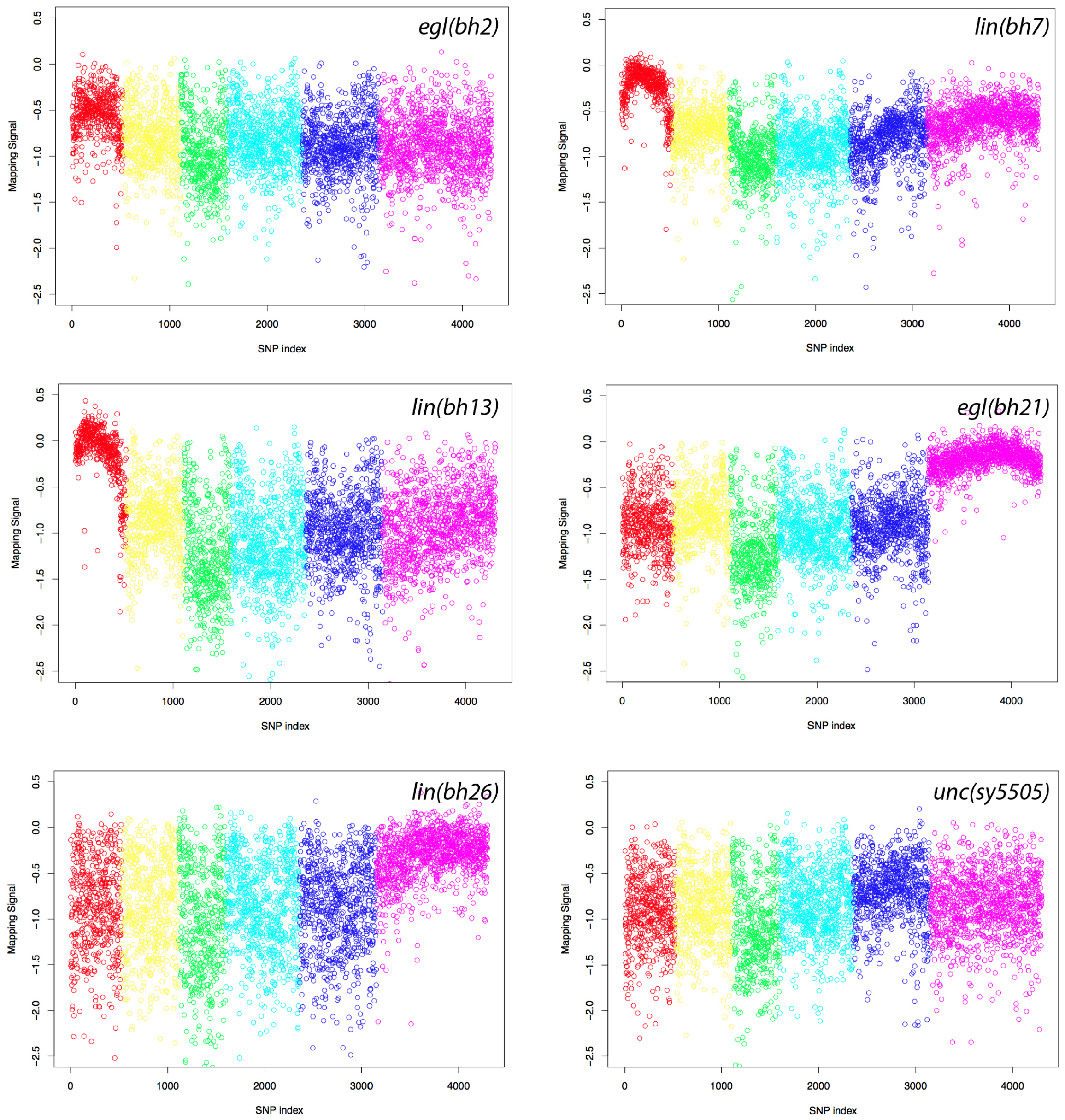

Figure S2 Mutation mapping using SNP-chip. Each tiny colored circle represents the mapping signal for a single SNP. Chromosomes are color coded (starting from 1 to 5 and X, in that order). See Materials and Methods and Zhao et al. study (ZHAO *et al.* 2010) for details.

Cbr-lin-11 cDNA sequence (1239 bp)

1	ATGCATTCTT	CTTCGTCCAT	CATCACCACC	CTGGAAGAAG	AAGAGAAGAA	GCCTCCTGCT
61	CATCTTCATC	AATATCATCA	TCATCTTCAT	CAACAGTCAG	TAGAAGACGT	CGGAAGTGCC
121	ACCTCATCAG	CCACGCTGCT	TCTTCTGGAT	ACTTCCGCTG	CCACGTGGAT	GATGCCGTCC
181	TCGACGACGC	ACCCTCAAAT	CTCCGAGATA	AGCGGAAATG	AATGCGCTGC	ATGTGCACAG
241	CCTATTCTTG	ACAGATATGT	ATTCACCGTT	CTTGGCAAAT	GTTGGCATCA	ATCATGTCTC
301	CGATGTTGCG	ATTGTCGAGC	TCCAATGTCG	ATGACTTGTT	TCAGTAAAGA	TGGCCTGATA
361	TTGTGTAAAA	CAGACTATTC	AAGAAGGTAC	GGTCATCGAT	GCGCTGGATG	TGATGGAAAA
421	CTGGAAAAAG	AGGATTTAGT	AAGGAGAGCA	AGAGACAAAG	TATTTCATAT	TCGATGTTTT
481	CAATGTTCAG	TATGTCAAAG	GCTCTTGGAT	ACGGGTGATC	AGCTTTATAT	CATGGAGGGA
541	AATCGATTCA	TGTGTCAAAA	TGATTTTCAA	ACGGCTACCA	AAACATCGAC	TCCAACATCA
601	ATGCACCGTC	CAATATCCAA	TGGATCCGAA	TGTAATTCCG	ATATCGAGGA	AGATAACGTG
661	GATGCTTGTG	ACGAGGGTGG	TCTTGACGAC	GTTGATGGTG	ACTGTGGAAA	GGATAACTCT
721	GATGACTCAA	ACTCTGCAAA	ACGGCGGGGT	CCTCGAACAA	CAATCAAAGC	TAAACAGCTT
781	GAAACATTGA	AAAATGCATT	CGCTGCGACC	CCGAAACCAA	CTCGACACAT	CCGTGAACAA
841	CTTGCTGCCG	AGACAGGGCT	GAACATGAGA	GTCATTCAGG	TGTGGTTCCA	AAATCGACGA
901	AGCAAGGAAC	GTCGAATGAA	ACAGCTTCGA	TACGGTGGAT	ATCGTCAATC	CAGAAGACAA
961	CGTCGAGAGG	ATATCGTTGA	TATGTTTCCG	AATGACCAAC	AGTTCTACCC	TCCACCACCT
1021	CCATCAAACG	TTCAATTCTT	CTGTGACCCA	TATGGAAGTC	CTCCAAATAA	CGGAGAGTCG
1081	ATGCAAATTC	CATCACAATT	CACAGTACCT	CCGGAGACTA	TGAATATGGT	GCCAGAACCA
1141	TATGCCGAAT	CATCGTCAAC	ACCACCAGAG	TTCAATGAAG	ATGCATTCAC	ATGCATTTAT
1201	TCCACTGATG	TCGGAAAACC	AACTCCAGTT	TCATGGTAG		

Exon		
	1: 254 bp	(1 - 254)
	2: 129 bp	(255 - 383)
	3: 139 bp	(384 - 522)
	4: 91 bp	(523 - 613)
	5: 62 bp	(614 - 675)
	6: 102 bp	(676 - 777)
	7: 102 bp	(778 - 879)
	8: 115 bp	(880 - 994)
	9: 141 bp	(995 - 1135)
	10: 104 bp	(1136 - 1239)

Figure S3 *Cbr-lin-11* cDNA sequence. The positions of exon-intron boundaries are marked.

Table S1 List of PCR and sequencing primers used in this study.

Oligo	Sequence (5' to 3')
GL380	GCTTCCCAATTCTCTGAGACGTCACA
GL381	GCCAAATTGCACAATTCCAGTTCAG
GL382	GTCCCGTTGAGACACACTTACATTG
GL383	CTCTGCTAGCTCCGACCACATTTC
GL384	AGGCTACTGTAGTTCTCATTTTAGGACCTA
GL385	GGTCAAAGCTAGAAGCCTATTAGAGCG
GL389	TTACGGTATTGGACGCCTAGGTAACC
GL390	GAGAAGTTCACACCTGCTGAGCTAC
GL391	GCAACAATGGAGCATCTACAGTAAGATCCC
GL392	AACAGGATACTGTGGTCCTGCTCCAATC
GL793	AGCTTCACATCTTGGTTCG
GL795	AAGAAACTCTGGATGGGCTC
GL800	CGGGAAGTTTGTGGAACG
GL801	GACAGAGTGCAGGAACAGC
GL802	GGCTCTCCTAATACATTCACG
GL806	TCCGAATAAGCGTAGGAGAC
GL807	AGCAGTCACTGTCCTTCC
GL808	AGATCCGTGTTGTCCAAGG
GL809	AGCTTCCTTGTCTACGGTC
GL810	GAGGTAGCGCCCAATTTATG
GL812	CCTCTATTCCAGCCAGAAACC
cb-lin-11-up-1	CCATGCATTCTTCTTCGTCCATCATCAC
cb-lin-11-up-2	TCTTCTGCAGTTCCGTTCTCGTTCATTTTCC
cb-lin-11-up-4	GGAAGTCCTCCAAATAACGGAGAG
cb-lin-11-up-5	GTCTTGACGACGTTGATGGTGACTGTG
cb-lin-11-up-6	GGAAATGAATGCGCTGCATGTGCAC
cb-lin-11-up-7	GATTCTACCATCTTTCCCACGCTGTAG
cb-lin-11-up-8	CTGGCTCTCCTCATCTAACTG
cb-lin-11-up-9	CTGGTCAATCGTACAGGGTTC
cb-lin-11-down-1	CCCTGCAGTGAAACTGGAGTTGGTTTTCC
cb-lin-11-down-2	GTGATGATGGACGAAGAAGAATGCATG
cb-lin-11-down-5	CTGAAAATGAAATGACTGGTCGGAGGG
cb-lin-11-down-7	GAACCCTGTGTGTCTCCTCTACTTC
cb-lin-11-down-8	GATAACTGACTCCAAATAGACGTAGGC
