# Supplementary material for: Genetic Control of Vulval Development in Caenorhabditis briggsae
Source: G3 (Bethesda). 2012 Dec 1;2(12):1625–41. doi: 10.1534/g3.112.004598 (PMC3516484; doi:10.1534/g3.112.004598)
Supplement: Supporting Information [file supp_2.12.1625_FigureS1.pdf]

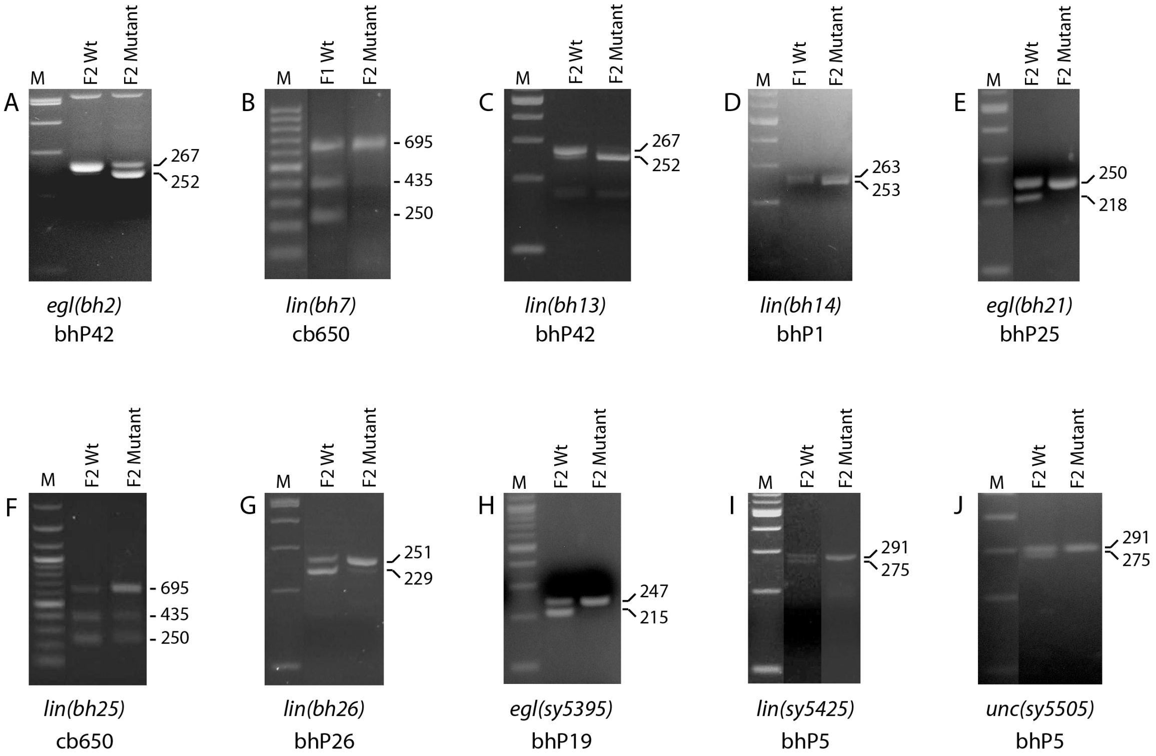

Figure S1 Mutation mapping using indel and snip-SNP polymorphisms. The images show DNA bands on 4% (for indels) and 1% (for snip-SNPs) agarose gels. The polymorphisms have been previously described (Koboldt *et al.* 2010). See Materials and Methods and Table 3 for details. M: DNA molecular weight marker, F1 Wt: DNA from F1 heterozygous animals, F2 Wt: DNA from phenotypically wild type pool of F2 animals, F2 Mutant: DNA from phenotypically mutant pool of F2 animals.
