# Supplementary material for: Genetic Control of Vulval Development in Caenorhabditis briggsae
Source: G3 (Bethesda). 2012 Dec 1;2(12):1625–41. doi: 10.1534/g3.112.004598 (PMC3516484; doi:10.1534/g3.112.004598)
Supplement: Supporting Information [file supp_2.12.1625_FigureS2.pdf]

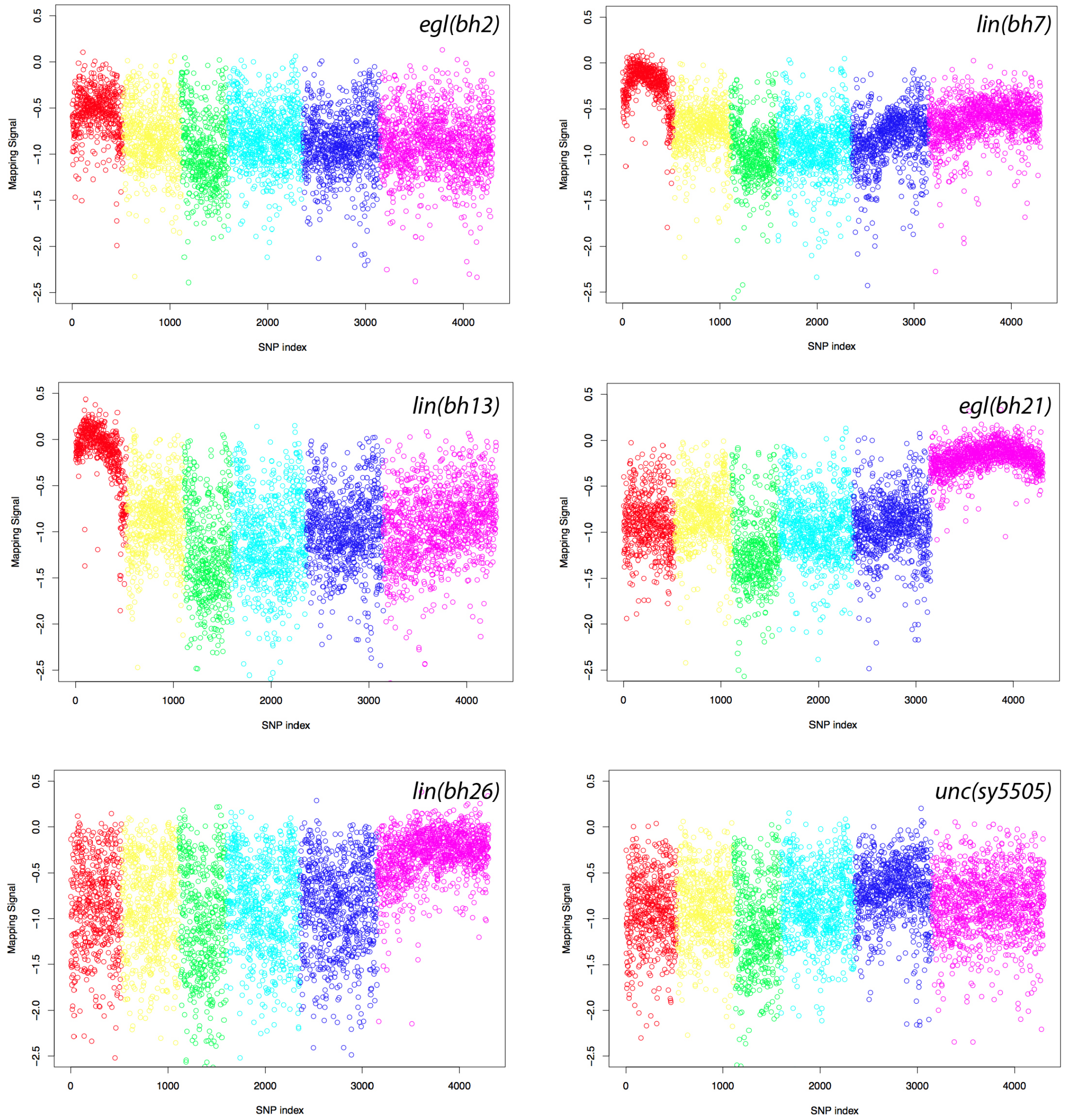

Figure S2 Mutation mapping using SNP-chip. Each tiny colored circle represents the mapping signal for a single SNP. Chromosomes are color coded (starting from 1 to 5 and X, in that order). See Materials and Methods and Zhao et al. study (ZHAO *et al.* 2010) for details.
