# Supplementary material for: Genetic Control of Vulval Development in Caenorhabditis briggsae
Source: G3 (Bethesda). 2012 Dec 1;2(12):1625–41. doi: 10.1534/g3.112.004598 (PMC3516484; doi:10.1534/g3.112.004598)
Supplement: Supporting Information [file supp_2.12.1625_FigureS3.pdf]

Cbr-lin-11 cDNA sequence (1239 bp)

```
   1 ATGCATTCTT CTTCGTCCAT CATCACCACC CTGGAAGAAG AAGAGAAGAA GCCTCCTGCT
  61 CATCTTCATC AATATCATCA TCATCTTCAT CAACAGTCAG TAGAAGACGT CGGAAGTGCC
 121 ACCTCATCAG CCACGCTGCT TCTTCTGGAT ACTTCCGCTG CCACGTGGAT GATGCCGTCC
 181 TCGACGACGC ACCCTCAAAT CTCCGAGATA AGCGGAAATG AATGCGCTGC ATGTGCACAG
 241 CCTATTCTTG ACAGATATGT ATTCACCGTT CTTGGCAAAT GTTGGCATCA ATCATGTCTC
 301 CGATGTTGCG ATTGTCGAGC TCCAATGTCG ATGACTTGTT TCAGTAAAGA TGGCCTGATA
 361 TTGTGTAAAA CAGACTATTC AAGAAGGTAC GGTCATCGAT GCGCTGGATG TGATGGAAAA
 421 CTGGAAAAAG AGGATTTAGT AAGGAGAGCA AGAGACAAAG TATTTCATAT TCGATGTTTT
 481 CAATGTTCAG TATGTCAAAG GCTCTTGGAT ACGGGTGATC AGCTTTATAT CATGGAGGGA
 541 AATCGATTCA TGTGTCAAAA TGATTTTCAA ACGGCTACCA AAACATCGAC TCCAACATCA
 601 ATGCACCGTC CAATATCCAA TGGATCCGAA TGTAATTCCG ATATCGAGGA AGATAACGTG
 661 GATGCTTGTG ACGAGGGTGG TCTTGACGAC GTTGATGGTG ACTGTGGAAA GGATAACTCT
 721 GATGACTCAA ACTCTGCAAA ACGGCGGGGT CCTCGAACAA CAATCAAAGC TAAACAGCTT
 781 GAAACATTGA AAAATGCATT CGCTGCGACC CCGAAACCAA CTCGACACAT CCGTGAACAA
 841 CTTGCTGCCG AGACAGGGCT GAACATGAGA GTCATTCAGG TGTGGTTCCA AAATCGACGA
 901 AGCAAGGAAC GTCGAATGAA ACAGCTTCGA TACGGTGGAT ATCGTCAATC CAGAAGACAA
 961 CGTCGAGAGG ATATCGTTGA TATGTTTCCG AATGACCAAC AGTTCTACCC TCCACCACCT
1021 CCATCAAACG TTCAATTCTT CTGTGACCCA TATGGAAGTC CTCCAAATAA CGGAGAGTCG
1081 ATGCAAATTC CATCACAATT CACAGTACCT CCGGAGACTA TGAATATGGT GCCAGAACCA
1141 TATGCCGAAT CATCGTCAAC ACCACCAGAG TTCAATGAAG ATGCATTCAC ATGCATTTAT
1201 TCCACTGATG TCGGAAAACC AACTCCAGTT TCATGGTAG
```

Exon	1: 254 bp	(1 - 254)
	2: 129 bp	(255 - 383)
	3: 139 bp	(384 - 522)
	4: 91 bp	(523 - 613)
	5: 62 bp	(614 - 675)
	6: 102 bp	(676 - 777)
	7: 102 bp	(778 - 879)
	8: 115 bp	(880 - 994)
	9: 141 bp	(995 - 1135)
	10: 104 bp	(1136 - 1239)

Figure S3 *Cbr-lin-11* cDNA sequence. The positions of exon-intron boundaries are marked.
