# Supplementary material for: Genetic Control of Vulval Development in Caenorhabditis briggsae
Source: G3 (Bethesda). 2012 Dec 1;2(12):1625–41. doi: 10.1534/g3.112.004598 (PMC3516484; doi:10.1534/g3.112.004598)
Supplement: Supporting Information [file supp_2.12.1625_TableS1.pdf]

Table S1 List of PCR and sequencing primers used in this study.

Oligo	Sequence (5’ to 3’)
GL380	GCTTCCCAATTCTCTGAGACGTCACA
GL381	GCCAAATTGCACAATTCCAGTTCAG
GL382	GTCCCGTTGAGACACACTTACATTG
GL383	CTCTGCTAGCTCCGACCACATTTC
GL384	AGGCTACTGTAGTTCTCATTTTAGGACCTA
GL385	GGTCAAAGCTAGAAGCCTATTAGAGCG
GL389	TTACGGTATTGGACGCCTAGGTAACC
GL390	GAGAAGTTCACACCTGCTGAGCTAC
GL391	GCAACAATGGAGCATCTACAGTAAGATCCC
GL392	AACAGGATACTGTGGTCCTGCTCCAATC
GL793	AGCTTCACATCTTGGTTCG
GL795	AAGAAACTCTGGATGGGCTC
GL800	CGGGAAGTTTGTGGAACG
GL801	GACAGAGTGCAGGAACAGC
GL802	GGCTCTCCTAATACATTCACG
GL806	TCCGAATAAGCGTAGGAGAC
GL807	AGCAGTCACTGTCCTTCC
GL808	AGATCCGTGTTGTCCAAGG
GL809	AGCTTCCTTGTCTACGGTC
GL810	GAGGTAGCGCCCAATTTATG
GL812	CCTCTATTCCAGCCAGAAACC
cb-lin-11-up-1	CCATGCATTCTTCTTCGTCCATCATCAC
cb-lin-11-up-2	TCTTCTGCAGTTCCGTTCTCGTTCATTTTCC
cb-lin-11-up-4	GGAAGTCCTCCAAATAACGGAGAG
cb-lin-11-up-5	GTCTTGACGACGTTGATGGTGACTGTG
cb-lin-11-up-6	GGAAATGAATGCGCTGCATGTGCAC
cb-lin-11-up-7	GATTCTACCATCTTTCCCACGCTGTAG
cb-lin-11-up-8	CTGGCTCTCCTCATCTAACTG
cb-lin-11-up-9	CTGGTCAATCGTACAGGGTTC
cb-lin-11-down-1	CCCTGCAGTGAAACTGGAGTTGGTTTTCC
cb-lin-11-down-2	GTGATGATGGACGAAGAAGAATGCATG
cb-lin-11-down-5	CTGAAAATGAAATGACTGGTCGGAGGG
cb-lin-11-down-7	GAACCCTGTGTGTCTCCTCTACTTC
cb-lin-11-down-8	GATAACTGACTCCAAATAGACGTAGGC
